# Research on modeling joining and joining modification method of hybrid FE-SEA model

**DOI:** 10.1038/s41598-023-43250-3

**Published:** 2023-09-22

**Authors:** Jintao Su, Ling Zheng, Jinquan Nie

**Affiliations:** 1 Hubei Key Laboratory of Power System Design and Test for Electrical Vehicle, Hubei University of Arts and Science, Xiangyang, 441053 China; 2 School of Automotive and Traffic Engineering, Hubei University of Arts and Science, Xiangyang, 441053 China; 3 Chongqing University, Chongqing, 401122 China

**Keywords:** Engineering, Mathematics and computing

## Abstract

Since the connection method at the boundary of the hybrid FE-SEA (finite element-statistical energy analysis, FE-SEA) model affects the overall calculation accuracy of the model, the missing or inaccurate connection relation will lead to a large error in the model calculation. In order to effectively solve the calculation error caused by the connection problem of hybrid FE-SEA model, this paper starts from the modeling methods and connection relations of “hybrid-point” connection, “hybrid-line” connection and “hybrid-surface” connection. In order to solve the modeling method and correction problems of the “hybrid-point” connection in the hybrid FE-SEA model, the “out-of-plane” wave motion equation at the “hybrid-point” connection was established by using the superposition principle of plane waveforms in polar coordinate system. The “hybrid-point” connection and wave-number relation in Cartesian coordinate system are studied. The radiation radius correction method of “hybrid-point” connection is proposed. An example is given to verify the effectiveness of the method. In order to solve the modeling and correction problem of “hybrid-line” in hybrid model, a “hybrid-line” connected triangular waveform function model was established by using the method of linear difference. The direct field dynamic stiffness matrix of “hybrid-line” connection in node coordinate system is studied. According to the shape function of the “hybrid-line” connection wavenumber space, a method to correct the shape function of the “hybrid-line” connection is proposed, and the validity of the method is verified.

## Introduction

The theory of classical statistical energy analysis has been extended in recent years to deal with acoustic vibration problems of non-conservative coupled systems, strongly coupled systems and indirectly coupled systems^1–3^. In addition, in the research field of classical statistical energy analysis, non-uniform mode energy statistical energy analysis, energy distribution, low mode density and non-resonant response, model variance prediction, extended method of statistical energy analysis, transient statistical energy analysis and other methods have also been further developed^4–8^. The classical statistical energy analysis model has certain advantages in solving high-frequency acoustic-vibration coupling problems due to its small computation scale^9–11^. However, in solving the intermediate frequency (1000–4000 Hz) acoustic problem, the calculation error is large. It is particularly important to find a solution to the intermediate frequency problem^12–14^.

FE-SEA hybrid method combines finite element and statistical energy methods. This method is an effective method to solve the coupling problem of medium frequency acoustic vibration. For parameter identification of mixed FE-SEA model, scholars have proposed some methods to obtain parameters of mixed FE-SEA model, but there are some shortcomings. Such as parameter stability, structural adaptability, etc. In addition, with the rapid increase of the number of subsystems, the calculation accuracy decreases. According to the development of the coupling problem and the classical SEA method, an accurate and feasible hybrid model parameter identification method is needed.

The current hybrid FE-SEA model is based on the infinite thin plate structure as the basic assumption. There are few literatures on the influence of connection boundary on point connection and the theory and method of hybrid point connection modeling. In the connection method research of hybrid model, Langley^15^ established the point connection model of infinite plate and beam structure based on wave theory. The coupling characteristics of point connection between elastic wave transmission coefficient and coupling loss factor are calculated. The general solution to the problem of point connection model is derived. However, the derivation process is based on the assumption of infinite structure, which has some limitations for the application of complex structure. Zhang^16^ extended and supplemented the modeling method of point connection in the model. The extension method of point connection modeling is verified by experimental means. The shortcoming of this method is that it ignores the complex changes of the location and boundary of the connection in the actual structure and does not consider the influence of the connection boundary on the modeling method of the point connection. Based on the concepts of hybrid finite element analysis and statistical energy analysis, Yong^17^ proposed a hybrid method for calculating the intermediate frequency vibration of a thin rectangular plate connected by a point and a line. In this method, the vibration of deterministic plates is described by the similarity analysis wave propagation method. By applying displacement continuity and force balance at the interface of point connection, the dynamic coupling between deterministic plate components and statistical plate components is established. On this basis, a hybrid formula for the intermediate frequency vibration of the thin plate system with point connection mode is proposed. The deterministic analytic wave of the thin plate component is described and the boundary condition limitation of the traditional analytic wave propagation method is eliminated. The numerical instability of the numerical wave propagation method is overcome. Yan^18^ proposed a beam-plate composite structure with hybrid point connection. The energy response of the composite structure was analyzed, and Monte Carlo method was used to check the hybrid FE-SEA model connection method. The results show that the low frequency vibration response of the structure is in good agreement with the experimental results. However, the error of high frequency vibration response is large, and this method overcomes the strict limitation of traditional methods for large and complex structures with uncertain factors. To avoid the shortcoming of using finite element method or intermediate frequency statistical energy analysis method to solve complex vibration acoustic system. Cotoni^19^ studied the linear connection model. The direct field dynamic stiffness matrix and the shape function of the linear connection model in the nodal coordinate system are derived. A hybrid linear model is used to predict the steady-state response of a vibrating acoustic system with uncertain properties. Finite element method is applied to the deterministic modeling of the subsystems with long wave attributes, and SEA method is applied to the statistical modeling of the subsystems with short wave attributes. The hybrid line connection is applied between the two models. The hybrid connection model is systematically studied by this method, and the connection structure of the hybrid model is acoustically predicted by an example. Zhu^20^ established a dynamic stiffness correction factor for hybrid-model point connection based on the characteristics of acoustic waves at the hybrid-model junction. The influence of hybrid connection boundary can be effectively modeled by modifying the point connection model at the junction. This correction method takes into account the influence of connection boundary on the modeling of point connection in hybrid model, and is a reasonable correction method for the modeling of point connection in hybrid model, which has certain theoretical significance. Chen^21^ derived the coupling model of arc connection, broken line connection and other kinds of line connection according to the calculation theory of line connection coupling model. The coupling system models of broken line connection, straight line connection, arc connection and arbitrary curve connection are established by numerical simulation. The coupling models of these four types of connection are numerically calculated, and the computational expressions of various line connections are derived theoretically. Zhu^22^ proposed a hybrid FE-SEA line connection modeling method based on Fourier transform theory. Based on wave theory, the impedance matrix of hybrid line connection of thin plate structure is derived. Then, the triangular waveform function is constructed for the displacement of the hybrid line connection. Finally, the line connection model in the node coordinate system is established according to the Fourier transform theory. The line connection model carries out wave transmission through incident waves, transmitted waves and reflected waves of the interface. An example is given to verify the accuracy of the linear connection modeling method. It is of great significance to study the method of line connection modeling for hybrid model. Although the method of linear connection modeling theory is elaborated. However, only the simple thin plate structure was verified. Modeling of complex structures with multiple subsystems is not discussed. The connection modeling method and correction method in hybrid model are the key of hybrid model correction technology. However, there are many literatures on hybrid model application technology, mainly focusing on the application of FE-SEA hybrid model for acoustics and other performance prediction, while less attention is paid to the reverse correction model technology based on prediction accuracy. There are a variety of connection modes in the hybrid FE-SEA model, including hybrid point connection, hybrid line connection, hybrid surface connection, as well as point, line and surface connection between SEA subsystems. The modeling modification methods of different connection modes have great influence on the prediction accuracy of the model. Among them, the theoretical research on the hybrid point connection, the line connection method and the correction method are still just beginning, and the relevant theories at home and abroad are relatively not perfect. Therefore, it is necessary to systematically study the connection method and model modification method of hybrid FE-SEA model.

In order to effectively solve the calculation error caused by the connection problem of hybrid FE-SEA model, this paper starts from the modeling methods and connection relations of “hybrid-point” connection, “hybrid-line” connection and “hybrid-surface” connection. In order to solve the modeling method and correction problems of the “hybrid-point” connection in the hybrid FE-SEA model, the "out-of-plane" wave motion equation at the “hybrid-point” connection was established by using the superposition principle of plane waveforms in polar coordinate system.

In order to solve the modeling and correction problem of “hybrid-line” in hybrid model, a “hybrid-line” connected triangular waveform function model was established by using the method of linear difference. The direct field dynamic stiffness matrix of “hybrid-line” connection in node coordinate system is studied. According to the shape function of the "hybrid-line" connection wavenumber space, a method to correct the shape function of the "hybrid-line" connection is proposed, and the validity of the method is verified.

## Basic theory of hybrid models

### Wave theory

**Figure 1:**
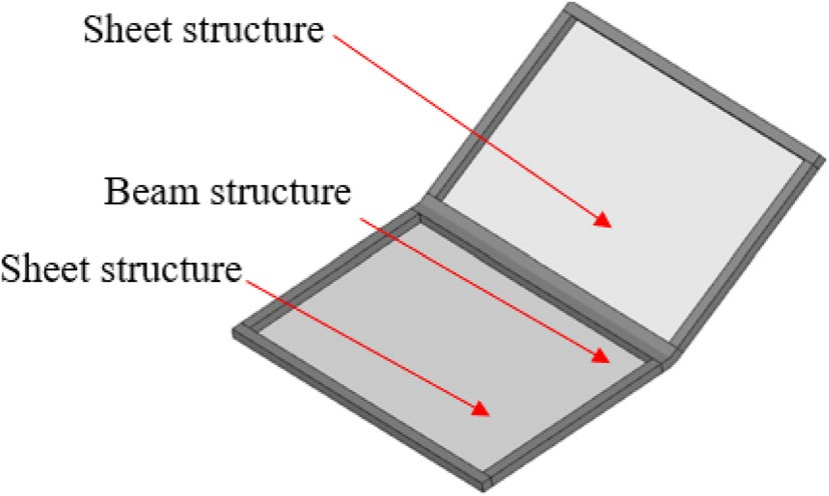
Simple composite system.

**Figure 2:**
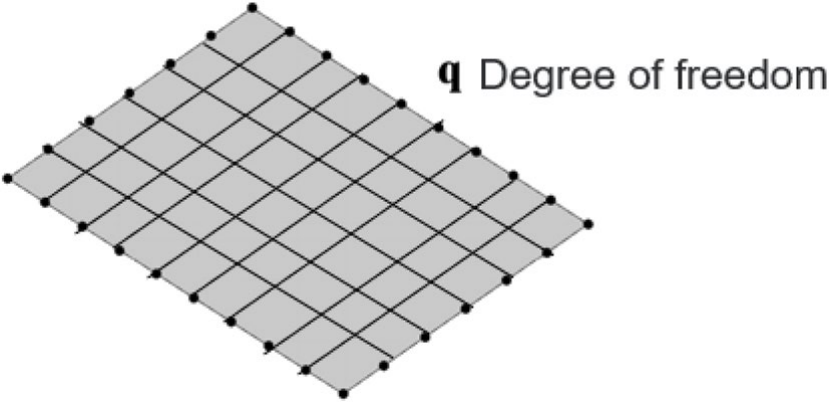
Boundary freedom.

According to the hybrid FE-SEA theory, the response of the stochastic subsystem is composed of the direct field and the reverberation field. The energy reciprocity principle exists between the direct field and reverberation field, and the average response of the subsystem can be solved by wave theory and modal method. The direct field is defined as the physical field in which the energy input to the subsystem does not pass through any boundary reflection. The reverberation field or diffusion field is defined as the physical field in which the energy input to the subsystem is reflected many times. The hybrid FE-SEA model carries out the system energy transfer and exchange between the “direct-reverberation field” through different connection methods. A simple composite system (consisting of two thin plates embedded in a beam) is used to describe the direct field-reverberation reciprocity relationship of the hybrid model system. As shown in Fig. 1, the most direct way to analyze the dynamic response of a system is to generate a finite element model. The degree of freedom consists of a set of displacements distributed throughout the system. Thin plate structures have relatively short vibration wavelengths, the idea to solve this problem can be realized by considering a single thin plate. As shown in Fig. 2, the displacement degree of freedom on the boundary is q. For harmonic motion of frequency $\omega$, the relationship between displacement degree of freedom on the boundary and a set of external forces f acting on the boundary can be expressed as follows:

1 $${\text{Dq}} = f$$

where *D* is the dynamic stiffness matrix, *q* is the displacement degree of freedom on the boundary, and f is the external force at the boundary. The dynamic characteristics of the whole system can be obtained from the dynamic characteristics of the composite beam frame of two thin plate structures in Fig. 1. The harmonic motion at the boundary is realized by wave theory to generate elastic waves. These elastic waves propagate throughout the system and bounce back each time a boundary is encountered.

The direct field dynamic stiffness matrix ${\text{D}}_{dir}$ is defined as the matrix *D* without reflection. The difference between the boundary force ${\text{D}}_{dir} q$ generated by this matrix and the actual boundary force *Dq* is called the “reverberation” force ${\text{f}}_{{{\text{rev}}}} {\text{ = D}}_{dir} q - Dq$. Then, Eq. (1) can be expressed as:

2 $${\text{D}}_{dir} q = f + {\text{f}}_{{{\text{rev}}}}$$

Assuming that the reverberation wave field is a “diffusion field”, then:

3 $$E[{\text{f}}_{{{\text{rev}}}} ] = 0$$

4 $$E[{\text{f}}_{{{\text{rev}}}} f_{rev}^{*T} ] = \left( {\frac{4E}{{\omega \pi n}}} \right){\text{Im}} \left\{ {{\text{D}}_{dir} } \right\}$$

where *E*[] represents the average value of the random structure, *E* is the vibration energy of the plate, and *n* is the modal density of the plate. Equation (4) is called “diffusion field reciprocity relation”. However, there are cases where components may be coupled to other components across domains, rather than just at the boundary, where a modal approach is needed for synthesis.

### Modal method

Based on modal theory, a modal parameter method is proposed. This method is used to describe the degree of freedom q of the thin plate in Figs. 1 and 2 as a subset of all the degrees of freedom. The direct field dynamic stiffness matrix ${\text{D}}_{dir}$ is replaced by the statistical mean of the dynamic stiffness matrix $E[D]$. The reverberation force vector ${\text{f}}_{{{\text{rev}}}}$ is replaced by a randomly varying force component $f_{ran}$. Therefore, $f_{ran} = E[D]q - Dq$, Eq. (2) can be expressed as^21^:

5 $$E[D]q = f + f_{ran}$$

Suppose, the degree of freedom q is at the component boundary, then the relation $E[D] = {\text{D}}_{dir}$ is obtained. In addition, the receiver matrix $H_{dir}$ is used to represent the inverse matrix of ${\text{D}}_{dir}$. According to these definitions, if the natural frequency and mode shape of the component conform to Gaussian orthogonality ensemble (GOE), then:

6 $$E[f_{ran} f_{ran}^{*T} ] = \left( {\frac{4E}{{\omega \pi n}}} \right){\text{Im}} \left\{ {{\text{D}}_{dir} } \right\} + \left( {\frac{2}{\pi m}} \right)[2{\text{Re}} ]\left\{ {S_{{\hat{f}\hat{f}}} } \right\} + q(m)S_{{\hat{f}\hat{f}}}$$

7 $$S_{{\hat{f}\hat{f}}} = E[\hat{f}\hat{f}^{*T} ]$$

8 $$\hat{f} = i{\text{D}}_{dir} {\text{Im}} \left\{ {{\text{H}}_{dir} } \right\}f$$

9 $$q(m) = - 1 + \left( {\frac{1}{2\pi m}} \right)(1 - e^{ - 2\pi m} ) + E_{1} (\pi m)\left[ {\cosh (\pi m) - \left( {\frac{1}{\pi m}} \right)\sinh (\pi m)} \right]$$

where $m = \omega \eta n$ represents the modal overlap of components, and $E_{1}$ represents the exponential integral.

## Hybrid FE-SEA modeling connection method

### Point connection of hybrid models

#### The equations of motion of point connection in hybrid model

**Figure 3:**
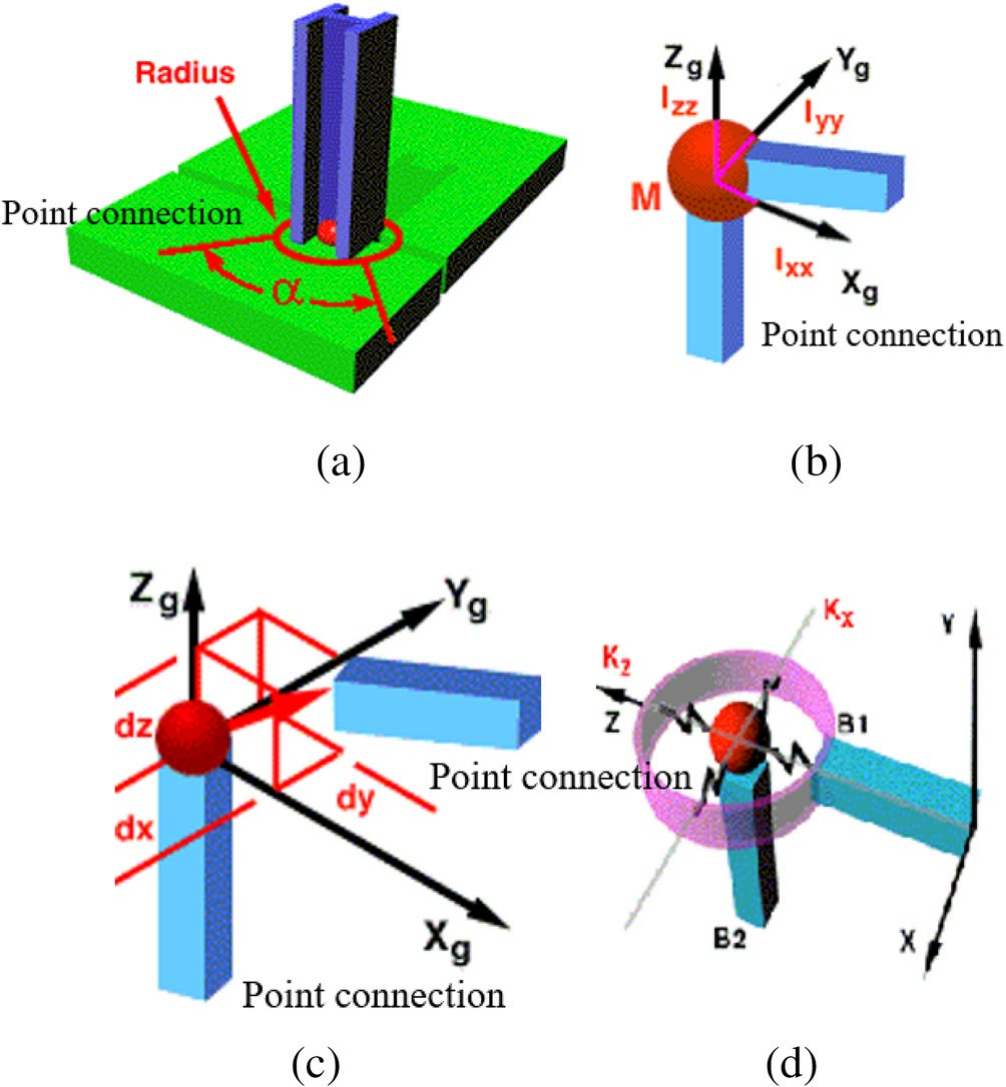
Schematic diagram of point connection relationship.

**Figure 4:**
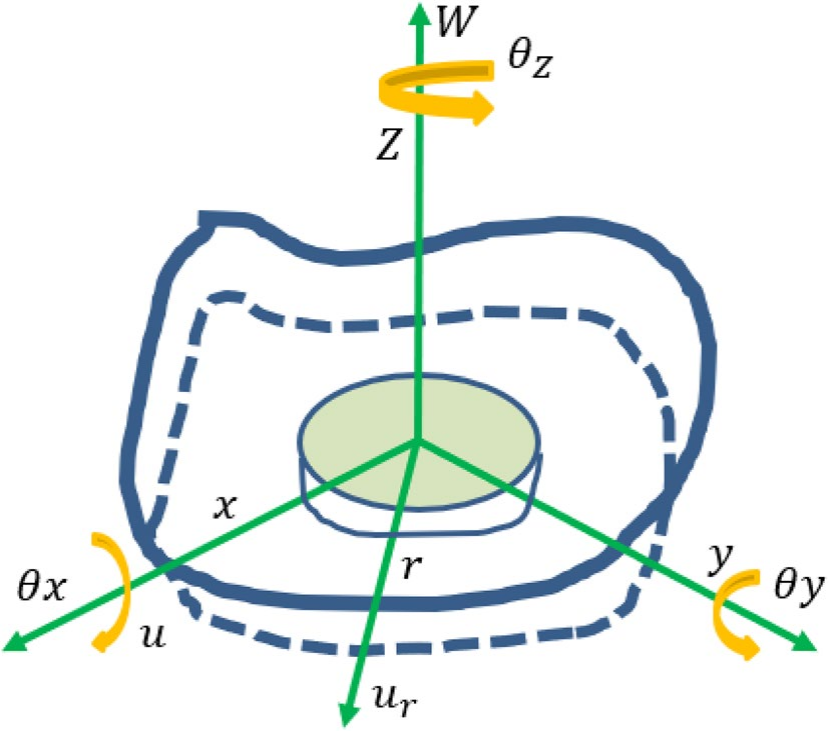
Points connect local coordinate systems.

Point connection is one of the important connection methods of hybrid model. Point connection generally exists between thin plate structure and beam structure. Energy transfer between hybrid models is carried out through nodes. Its connection relationship is shown in Fig. 3. Figure 3a shows the point connection relationship between plate and beam structure, and Angle α represents the radiation Angle of point connection. Figure 3b represents the point connection mass and moment of inertia of the two connection structures, and M represents the connection mass at the point connection. Xg, Yg and Zg respectively represent the coordinate system at the point connection, and Ixx, Iyy and Izz respectively represent the moment of inertia in three directions at the point connection. Figure 3c shows the point-connection offset distance of the two-connection structure. dx, dy and dz respectively represent the offset distance of the point connection structure on the three coordinate axes X, Y and Z. Figure 3d shows the stiffness effect of the point connection structure, and B1 and B2 represent two independent subsystems respectively. Kx and Kz represent the connection stiffness on the X axis and Z axis respectively. The local coordinate system of point connection is established, as shown in Fig. 4. It is assumed that the point connection mode of infinite thin plates is a massless rigid circular plane. The infinite thin plate contains three kinds of propagation waveform and three kinds of dissipation waveform: plane displacement wave $\omega$, plane bending wave $\theta_{x}$ and plane bending wave $\theta_{y}$. And the tensile and shear waves associated with the in-plane displacements $u$ and $\upsilon$. And the shear wave associated with the Angle $\theta_{z}$. Under the condition of polar coordinates $(r,\theta ,z)$ with point connection as the origin, the out-of-plane displacement of an infinite thin plate can be expressed as the superposition of six waveforms related to $\omega$, $\theta_{x}$ and $\theta_{y}$, then it can be deduced^6^ :

10 $$\begin{aligned} & u_{z} (r,\theta ) = a_{0}^{(b)} H_{0}^{(2)} (k_{b} r) + a_{1s}^{(b)} H_{1}^{(2)} (k_{b} r)\sin \theta + a_{1c}^{(b)} H_{1}^{(2)} (k_{b} r)\cos \theta \\ & \quad + a_{0}^{(bn)} H_{0}^{(2)} ( - ik_{b} r) + a_{1s}^{(bn)} H_{1}^{(2)} ( - ik_{b} r)\sin \theta + a_{1c}^{(bn)} H_{1}^{(2)} ( - ik_{b} r)\cos \theta \\ \end{aligned}$$

where $u_{z}$ is the plane external displacement of the thin plate in the polar coordinate system, and $H_{{\text{n}}}^{(2)}$ is the Hankel function. $a_{0}^{(b)}$, $a_{1s}^{(b)}$ and $a_{1c}^{(b)}$ are the amplitudes of the propagation waveform associated with $(\omega ,\theta_{x} ,\theta_{y} )$ respectively.$a_{0}^{(bn)}$, $a_{1s}^{(bn)}$ and $a_{1c}^{(bn)}$ are the amplitudes of the dissipative wavelength respectively. The wave number in the bending direction of the sheet is $k_{b}$. The in-plane motion of a thin plate can be described as a tensile wave in-plane and a shear wave in-plane, and the relationship is as follows^20^:

11 $$\begin{aligned} & u_{r} (r,\theta ) = a_{1c}^{(e)} [H_{0}^{(2)} (k_{e} r) - \frac{{H_{1}^{(2)} (k_{e} r)}}{{k_{e} r}}]\cos (\theta ) + a_{1s}^{(e)} [H_{0}^{(2)} (k_{e} r) - \frac{{H_{1}^{(2)} (k_{e} r)}}{{k_{e} r}}]\cos (\theta ) \\ & \quad + \frac{1}{{k_{s} r}}[a_{1s}^{(s)} H_{1}^{(2)} (k_{s} r)\cos (\theta ) - a_{1c}^{(s)} H_{1}^{(2)} (k_{s} r)\sin (\theta )] \\ \end{aligned}$$

12 $$\begin{aligned} & u_{\theta } (r,\theta ) = \frac{1}{{k_{e} r}}[a_{1s}^{(e)} H_{1}^{(2)} (k_{e} r)\cos (\theta ) - a_{1c}^{(e)} H_{1}^{(2)} (k_{e} r)\sin (\theta )] + a_{0}^{(s)} H_{1}^{(2)} (k_{s} r) \\ & \quad - a_{1c}^{(s)} [H_{0}^{(2)} (k_{s} r) - \frac{{H_{1}^{(2)} (k_{s} r)}}{{k_{s} r}}]\cos (\theta ) - a_{1s}^{(s)} [H_{0}^{(2)} (k_{s} r) - \frac{{H_{1}^{(2)} (k_{s} r)}}{{k_{s} r}}]\sin (\theta ) \\ \end{aligned}$$

where $a_{1c}^{(e)}$ and $a_{1s}^{(e)}$ are the amplitudes of the plane tensile propagation waveform related to $u$ and $\upsilon$ respectively. $a_{0}^{(s)}$ is the amplitude of the shear plane wave in-plane. $a_{1c}^{(s)}$ and $a_{1s}^{(s)}$ are the amplitudes of the plane shear waveforms associated with $u$ and $\upsilon$, respectively. $k_{e}$ and $k_{s}$ are respectively the tensile and shear wave numbers of the thin plate.

#### The relationship between point connection and wave number in hybrid model

According to the bending theory of circular thin plate, the expressions of bending moment ${\text{M}}_{{\text{x}}}$, ${\text{M}}_{{\text{y}}}$ and external force $F_{z}$ in Cartesian coordinate system are obtained as follows^21^:

13 $${\text{M}}_{{\text{x}}} = r\int\limits_{0}^{2\pi } {\{ rD[\frac{{\partial^{3} u_{z} }}{{\partial r^{3} }} + \frac{1}{r}\frac{{\partial^{2} u_{z} }}{{\partial r^{2} }} - \frac{1}{{r^{2} }}\frac{{\partial u_{z} }}{\partial r} - \frac{(3 - \upsilon )}{{r^{3} }}\frac{{\partial^{2} u_{z} }}{{\partial \theta^{2} }} + \frac{(2 - \upsilon )}{{r^{2} }}\frac{{\partial^{3} u_{z} }}{{\partial r\partial \theta^{2} }}] - D(\frac{{\partial^{2} u_{z} }}{{\partial r^{2} }} + \frac{\upsilon }{{r^{2} }}\frac{{\partial^{2} u_{z} }}{{\partial \theta^{2} }} + \frac{\upsilon }{r}\frac{{\partial u_{z} }}{\partial r})\} \sin \theta d\theta }$$

14 $${\text{M}}_{{\text{y}}} = - r\int\limits_{0}^{2\pi } {\{ rD[\frac{{\partial^{3} u_{z} }}{{\partial r^{3} }} + \frac{1}{r}\frac{{\partial^{2} u_{z} }}{{\partial r^{2} }} - \frac{1}{{r^{2} }}\frac{{\partial u_{z} }}{\partial r} - \frac{(3 - \upsilon )}{{r^{3} }}\frac{{\partial^{2} u_{z} }}{{\partial \theta^{2} }} + \frac{(2 - \upsilon )}{{r^{2} }}\frac{{\partial^{3} u_{z} }}{{\partial r\partial \theta^{2} }}] - } D(\frac{{\partial^{2} u_{z} }}{{\partial r^{2} }} + \frac{\upsilon }{{r^{2} }}\frac{{\partial^{2} u_{z} }}{{\partial \theta^{2} }} + \frac{\upsilon }{r}\frac{{\partial u_{z} }}{\partial r})\} \cos \theta d\theta$$

15 $${\text{F}}_{{\text{z}}} = r\int\limits_{0}^{2\pi } {D[\frac{{\partial^{3} u_{z} }}{{\partial r^{3} }} + \frac{1}{r}\frac{{\partial^{2} u_{z} }}{{\partial r^{2} }} - \frac{1}{{r^{2} }}\frac{{\partial u_{z} }}{\partial r} - ...\frac{(3 - \upsilon )}{{r^{3} }}\frac{{\partial^{2} u_{z} }}{{\partial \theta^{2} }} + \frac{(2 - \upsilon )}{{r^{2} }}\frac{{\partial^{3} u_{z} }}{{\partial r\partial \theta^{2} }}]d} \theta$$

where in the Cartesian coordinate system, the expressions of external forces $F_{x}$,$F_{y}$ and bending moment $M_{z}$ are as follows:

16 $$F_{x} = - hr\int\limits_{0}^{2\pi } {[\frac{E}{{1 - \upsilon^{2} }}} (\frac{{\partial u_{r} }}{\partial r} + \frac{\upsilon }{r}u_{r} + \frac{\upsilon }{r}\frac{{\partial u_{\theta } }}{\partial \theta })\cos \theta ... - G(\frac{1}{r}\frac{{\partial u_{r} }}{\partial \theta } - \frac{{u_{\theta } }}{r} + \frac{{\partial u_{\theta } }}{\partial r})\sin \theta ]d\theta$$

17 $$F_{y} = - hr\int\limits_{0}^{2\pi } {[\frac{E}{{1 - \upsilon^{2} }}} (\frac{{\partial u_{r} }}{\partial r} + \frac{\upsilon }{r}u_{r} + \frac{\upsilon }{r}\frac{{\partial u_{\theta } }}{\partial \theta })\sin \theta ... + G(\frac{1}{r}\frac{{\partial u_{r} }}{\partial \theta } - \frac{{u_{\theta } }}{r} + \frac{{\partial u_{\theta } }}{\partial r})\cos \theta ]d\theta$$

18 $$M_{z} = - hr^{2} \int\limits_{0}^{2\pi } {G(\frac{1}{r}\frac{{\partial u_{r} }}{\partial \theta } - \frac{{u_{\theta } }}{r} + \frac{{\partial u_{\theta } }}{\partial r})d\theta }$$

Substituting the above Eqs. (10), (11) and (12) into Eqs. (13) to (18), the displacement equation of thin plate structure expressed by waveform coefficient can be obtained as follows. Where S represents the transverse force.

19 $$F = Sa$$

### Line connection of hybrid models

**Figure 5:**
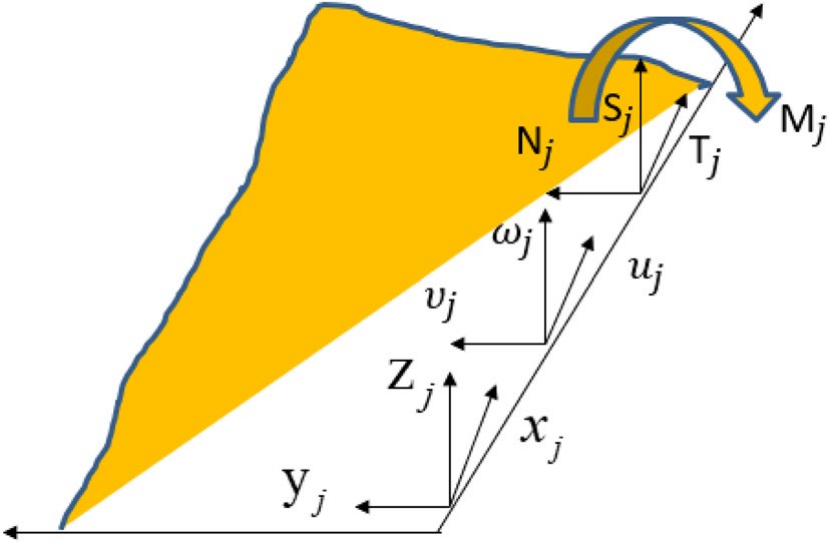
Local coordinate system of line connection mode.

**Figure 6:**
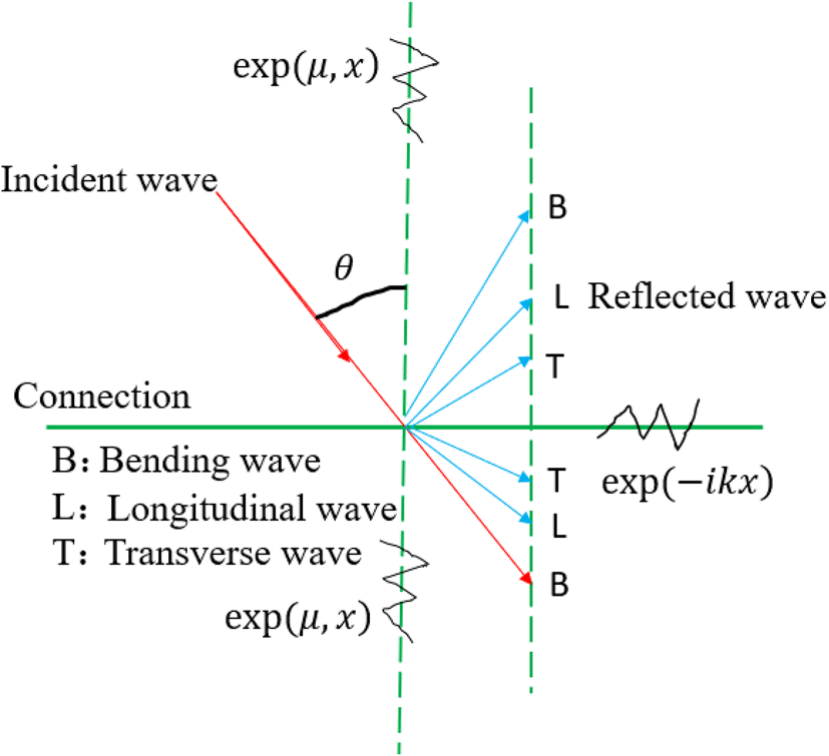
Snell's theorem for wire connections.

A line connection is a form of connection used to describe a thin plate component in a hybrid model. If the coupling between the structural subsystem and the acoustic cavity subsystem is not considered in the system, the connection mode of the system is mainly line connection and point connection. In general, the line connection mode describing the system is shown in Figs. 5 and 6. Figure 5 shows the local coordinate system ($x_{j}$, ${\text{y}}_{j}$, ${\text{z}}_{j}$) of the line connection of the $j$-block plate of the hybrid model. The connection is located at ($y_{j} = 0$). Assuming ($j$) is a thin plate, the equation of motion at the line junction can be described by translational motion in three directions ($(u_{j} ,\upsilon_{j} ,\omega_{j} )^{T}$) and rotation ($\theta_{j}$) about the X-axis. According to the isotropic theory of thin plate, the governing equation of transverse free motion of thin plate can be described as^22^:

20 $$D_{o} \nabla^{4} \omega + m\frac{{\partial^{2} \omega }}{{\partial t^{2} }} = 0$$

where, $\nabla^{4} = \left( {\frac{{\partial^{2} }}{{\partial x^{2} }} + \frac{{\partial^{2} }}{{\partial y^{2} }}} \right)^{2}$, $D_{o}$ is the bending stiffness of the thin plate. *m* is the mass per unit area. According to the Snell theorem (as shown in Fig. 6), the transmitted wave and the reflected wave have the same spatial wave term. The waveform of the structure at the junction line has the same trajectory velocity to satisfy the phase compatibility condition at the junction. Assuming that both the transmitted and reflected waves are bending waves, the wave term is $\exp ( - ikx + \mu_{B} y + i\omega t)$. Where $\mu_{B}$ is the undefined wavenumber in the y direction, and k is the wavenumber in the x direction. If the Angle between the incident wave and the normal is set as $\phi ^{\prime}$ and the wave number is $k_{B}^{\prime }$, then k can be expressed as^21^:

21 $$k = k_{B}{\prime} \sin \phi ^{\prime}$$

Substitute $\exp ( - ikx + \mu_{B} y + i\omega t)$ into Eq. (20) to get $\mu_{B}$. The following relationship is satisfied:

22 $$\mu_{B}^{2} = k^{2} \pm k_{B}^{2}$$

where $k_{B}$ is the bending wave number. If the two values of $\mu_{B}$ are $\mu_{B1}$ and $\mu_{B2}$ respectively, then the transverse displacement of the thin plate can be expressed as:

23 $$\omega = (a_{1} \exp (\mu_{B1} y) + a_{2} \exp (\mu_{B2} y))\exp ( - ikx + i\omega t)$$

The Angle $\theta_{x}$ in the x direction is expressed as $\theta_{x} = \frac{\partial \omega }{{\partial y}}$. Substitute it into Eq. (23), and the following relationship can be obtained:

24 $$\theta_{x} = (a_{1} \mu_{B1} \exp (\mu_{B1} y) + a_{2} \mu_{B2} y\exp (\mu_{B2} y))\exp ( - ikx + i\omega t)$$

Considering the boundary, y = 0, and Eqs. (23) and (24), the following relation can be obtained:

25 $$\left( {\begin{array}{*{20}c} \omega \\ {\theta_{x} } \\ \end{array} } \right)_{y = 0} = \left( {\begin{array}{*{20}c} 1 & 1 \\ {\mu_{B1} } & {\mu_{B2} } \\ \end{array} } \right)\left( {\begin{array}{*{20}c} {a_{1} } \\ {a_{2} } \\ \end{array} } \right)\exp ( - ikx + i\omega t)$$

By omitting $\exp ( - ikx + i\omega t)$, $a_{1}$ and $a_{2}$ can be obtained from Eq. (25) :

26 $$a_{1} = \frac{{\mu_{B2} \omega }}{{\mu_{B2} - \mu_{B1} }} - \frac{\theta }{{\mu_{B2} - \mu_{B1} }}$$

27 $$a_{2} = \frac{{ - \mu_{B1} \omega }}{{\mu_{B2} - \mu_{B1} }} + \frac{\theta }{{\mu_{B2} - \mu_{B1} }}$$

According to the classical thin shell plate theory, the X-axis moment and the transverse force can be expressed as:

28 $$M = D_{0} \left( {\frac{{\partial^{2} \omega }}{{\partial y^{2} }}{ + }\upsilon \frac{{\partial^{2} \omega }}{{\partial x^{2} }}} \right)_{y = 0}$$

29 $$S = - D_{0} (\frac{{\partial^{3} \omega }}{{\partial y^{3} }} + (2 - \upsilon )\frac{{\partial^{3} \omega }}{{\partial x^{2} \partial y}})_{y = 0}$$

where υ is the Poisson's ratio of thin plate structure. Substituting Eq. (23) into Eqs. (28) and (29), the following relation can be obtained^20^:

30 $${\text{M}} = {\text{D}}_{0} [(a_{1} \mu_{B1}^{2} + a_{2} \mu_{B2}^{2} ) - \upsilon (a_{1} + a_{2} )k^{2} ]$$

31 $$S = D_{0} [(a_{1} \mu_{B1}^{3} + a_{2} \mu_{B2}^{3} ) - (2 - \upsilon )(a_{1} \mu_{B1} + a_{2} \mu_{B2} )k^{2} ]$$

Substituting Eqs. (30) and (31) into Eqs. (26) and (27), the following relationship can be obtained:

32 $$\left[ {\begin{array}{*{20}c} S \\ M \\ \end{array} } \right] = D_{0} \left[ {\begin{array}{*{20}l} {\mu_{B1} \mu_{B2} (\mu_{B1} + \mu_{B2} )} \hfill & { - (\mu_{B1}^{2} + \mu_{B2}^{2} + \mu_{B1} \mu_{B2} ) + (2 - \upsilon )k^{2} )} \hfill \\ { - (\mu_{B1} \mu_{B2} + \upsilon k^{2} )} \hfill & {\mu_{B1} + \mu_{B2} } \hfill \\ \end{array} } \right].\left[ {\begin{array}{*{20}c} \omega \\ {\theta_{x} } \\ \end{array} } \right]$$

where $\mu_{B1}^{2} + \mu_{B2}^{2} = 2k^{2}$, the dynamic stiffness matrix of transverse vibration of thin plate line connection can be written as:

33 $${\text{D}}_{B} = D_{0} \left[ {\begin{array}{*{20}c} {\mu_{B1} \mu_{B2} (\mu_{B1} + \mu_{B2} )} & { - (\mu_{B1} \mu_{B2} + \upsilon k^{2} )} \\ { - (\mu_{B1} \mu_{B2} + \upsilon k^{2} )} & {\mu_{B1} + \mu_{B2} } \\ \end{array} } \right]$$

### Surface connection of hybrid models

**Figure 7:**
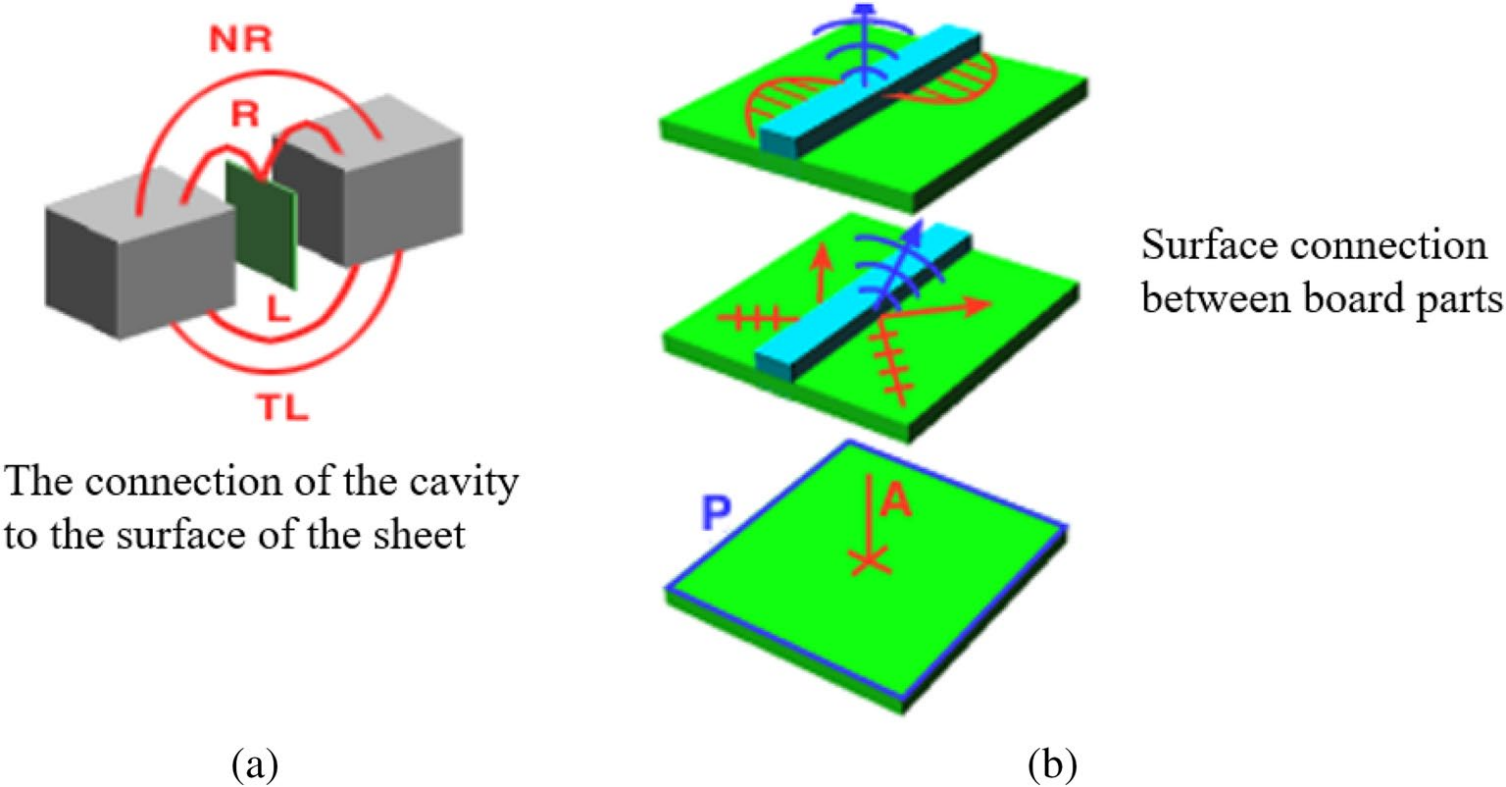
Schematic diagram of surface connection.

In the hybrid model, the boundary between the plate and the volume of the cavity is related to the out-of-plane displacement of the whole plate. This coupling connection mode is called “surface connection” or “area connection”, and the diagram of surface connection relationship is shown in Fig. 7. Figure 7a shows the diagram of surface connection between the thin plate and the two sound cavities, where L represents the distance between the two sound cavities. TL represents the acoustic transmission loss between two sound cavities. R represents the radiation efficiency between the thin plate structure and the two sound cavities. NR stands for noise emission between two cavities. Figure 7b shows a schematic diagram of the surface connection between the panels. The arrows in the figure represent the sound wave transmission at the interface between plates, P represents the thin plate structure, and A represents the sound wave of the thin plate structure. When the subsystems in the hybrid model have plane connections, the boundary degree of freedom Q can be defined as the out-of-plane response covering the point grid on the thin plate surface. In order to calculate the system response of Eq. (4), it is necessary to identify the dynamic stiffness matrix ${\text{D}}_{dir}$. In this case, the *jk* term of the receive matrix can be expressed as:

34 $${\text{H}}_{dir,jk} = {\text{G(r}}_{{{\text{jk}}}} {)}$$

where ${\text{r}}_{{{\text{jk}}}}$ is the distance from grid node J to K, and G is Green's function of an infinite plate. The dynamic stiffness matrix ${\text{D}}_{dir}$ is obtained by the inverse matrix of $H_{dir}$. Under the condition that the thin plate is directly excited, it can be deduced from Eq. (5) :

35 $${\text{q}} = {\text{H}}_{dir} f + {\text{H}}_{dir} f_{ran}$$

where the first term on the right side of the equation is the response of the infinite thin plate, and the second term is the additional response caused by the system. For weakly damped systems, the second term dominates. By combining Eq. (35) with Eq. (6), the following relation can be obtained^21^:

36 $${\text{S}}_{{{\text{qq}}}} = E[qq^{*T} ] = H_{dir} E[f_{ran} f_{ran}^{*T} ]H_{dir}^{*T} = \left( {\frac{4E}{{\pi \omega n}}} \right)H_{dir} {\text{Im}} \left\{ {D_{dir} } \right\}H_{dir}^{*T}$$

In Eq. (6), assuming that the second term on the right side of the equation can be ignored, the following matrix identity applies to any matrix A:

37 $$A{\text{Im}} \left\{ {A^{ - 1} } \right\}A^{*} = - {\text{Im}} \left\{ A \right\}$$

Therefore, Eq. (36) can be transformed into:

38 $${\text{S}}_{{{\text{qq}}}} = - \left( {\frac{4E}{{\pi \omega n}}} \right){\text{Im}} \left\{ {H_{dir} } \right\} \Rightarrow E[q_{j} q_{k}^{*} ] = - \left( {\frac{4E}{{\pi \omega n}}} \right){\text{Im}} \left\{ {{\text{G(r}}_{{{\text{jk}}}} {)}} \right\}$$

Equation (38) represents the correlation between the response at node j and the response at node k. It's proportional to the imaginary part of Green's function between two points. Based on the above equation, the system response at the hybrid surface joint can be determined. The degree of freedom in the hybrid model is composed of a free grid ${\text{q}}_{i}$ connected by each region. It is expressed as a deterministic set of degrees of freedom ${\text{q}}_{m}$ that forms associated components with deterministic finite element (FE) models. A particular collection ${\text{q}}_{i}$ has multiple SEA subsystems. The thin plate and the sound cavity share a common grid, so that the general structure of the equation has the form of a complete set of determined degrees of freedom^20–22^:

39 $$D_{d} q = \left( {\begin{array}{*{20}c} {D_{m} } & {C_{1} } & {C_{2} } & {...} \\ {C_{1}^{*T} } & {\sum\limits_{j = 1}^{N1} {D_{dir,j}^{1} } } & 0 & {...} \\ {C_{2}^{*T} } & 0 & {\sum\limits_{j = 1}^{N2} {D_{dir,j}^{2} } } & {...} \\ {...} & {...} & {...} & {...} \\ \end{array} } \right)\left( {\begin{array}{*{20}c} {q_{m} } \\ {q_{1} } \\ {q_{2} } \\ {...} \\ \end{array} } \right) = \left( {\begin{array}{*{20}c} {F_{m} } \\ {F_{1} } \\ {F_{2} } \\ {...} \\ \end{array} } \right)$$

where $D_{m}$ is the dynamic stiffness matrix of the (FE) model, including the “direct field” dynamic stiffness matrix of any subsystem connected to the model by non-regional nodes. $Ni$ is the number of subsystems connected to the grid of the i node, and $D_{dir,j}^{i}$ is the dynamic stiffness matrix of the jth subsystem connected to the ith grid node in the direct field. $C_{i}$ is the deterministic coupling matrix (assuming no power is consumed in the coupling, the coupling is the Hermitian matrix). $F_{i}$ represents the external force acting directly on $q_{i}$ degree of freedom and the reverberation force generated by the additional subsystem. One of the solving difficulties of the current method is that $q_{i}$ may contain multiple degrees of freedom, so it can be further reduced by directly applying the hybrid method in Eq. (39) to obtain:

40 $$\left( {\begin{array}{*{20}c} {D_{m} } & {C_{1} } \\ {C_{1}^{*T} } & {\sum\limits_{j = 1}^{N1} {D_{dir,j}^{i} } } \\ \end{array} } \right)\left( {\begin{array}{*{20}c} {q_{m} } \\ {q_{1} } \\ \end{array} } \right) = \left( {\begin{array}{*{20}c} {F_{m} } \\ {F_{1} } \\ \end{array} } \right)$$

Further, the matrices involved are reduced by rearrangement:

41 $$\left[ {D_{m} - C_{1} \left( {\sum\limits_{j = 1}^{N1} {D_{dir,j}^{i} } } \right)^{ - 1} D_{dir,j}^{i} C_{1}^{*T} } \right]q_{m} = F_{m} - C_{1} \left( {\sum\limits_{j = 1}^{N1} {D_{dir,j}^{i} } } \right)^{ - 1} F_{1}$$

42 $$D_{m.tot} = D_{m} - C_{1} D_{dir}^{1\quad - 1} C_{1}^{*T}$$

43 $$D_{dir}^{1} = \sum\limits_{j = 1}^{N1} {D_{dir,j}^{i} }$$

Therefore, Eq. (40) can be written as:

44 $$D_{m.tot} q_{m} = F_{m} - C_{1} D_{dir}^{1\quad - 1} F_{1}$$

Equation (44) is a simplified form of deterministic system equation. Where, the degree of freedom of $q_{1}$ does not reflect, and the energy flow generated by the reverberation field of subsystem j is considered. According to Eq. (44), the deterministic subsystem response $F_{1}$ is provided by the following equation:

45 $$q_{m} = - D_{m,tot}^{ - 1} C_{1} D_{dir}^{1\quad - 1} F_{1,j}$$

According to the principle of mutuality of the hybrid model, the following can be obtained^21^:

46 $$E[F_{1,j} F_{1,J}^{*T} ] = \left( {\frac{{4E_{j} }}{{\omega \pi n_{j} }}} \right){\text{Im}} \left\{ {D_{dir,j}^{1} } \right\}$$

47 $$S_{qq} = E[q_{m} q_{m}^{*T} ] = \left( {\frac{{4E_{j} }}{{\omega \pi n_{j} }}} \right)D_{m,tot}^{ - 1} C_{1} D_{dir}^{1\quad - 1} {\text{Im}} \left\{ {D_{dir,j}^{1} } \right\}D_{dir}^{1\quad - 1*T} C_{1}^{*T} D_{m,tot}^{ - 1*T}$$

The above results can be rewritten as:

48 $$S_{qq} = \left( {\frac{{4E_{j} }}{{\omega \pi n_{j} }}} \right)D_{m,tot}^{ - 1} {\text{Im}} \left\{ {D_{red,j}^{1} } \right\}D_{m,tot}^{ - 1*T}$$

where, the reduced direct field dynamic stiffness matrix is expressed as:

49 $$D_{red,j}^{1} = - C_{1} D_{dir}^{1\quad - 1} D_{dir,j}^{1*} D_{dir}^{1\quad - 1*T} C_{1}^{*T}$$

Assuming that the dynamic stiffness matrix is symmetric, it can be concluded that:

50 $$\sum\limits_{j = 1}^{N1} {D_{red,j}^{1} } = - C_{1} D_{dir}^{{1\quad - 1}{}} \left( {\sum\limits_{j = 1}^{N1} {D_{red,j}^{1*} } } \right)D_{dir}^{1\quad - 1*T} C_{1}^{*T} = - C_{1} D_{dir}^{1\quad - 1} C_{1}^{*T}$$

Therefore, the input power of subsystem k caused by the response of Eq. (48) is:

51 $${\text{P}}_{{\text{k}}} = (\omega /2)q_{1}^{{*{\text{T}}}} {\text{Im}} \left\{ {D_{dir,k}^{1} } \right\}q_{1}$$

52 $$q_{1} = D_{dir}^{1\quad - 1} C_{1}^{*T} q_{m}$$

According to Eq. (40) and the condition that $F_{1} = 0$, the power equation is:

53 $$P_{k} = \left( {\frac{{2E_{j} }}{{\pi n_{j} }}} \right)\sum\limits_{r,s} {\text{Im}} \left\{ {D_{red,k,rs}^{1} } \right\}(D_{m,tot}^{ - 1} {\text{Im}} \left\{ {D_{red,j}^{1} } \right\}D_{m,tot}^{ - 1*T} )_{rs}$$

According to the above method, the standard hybrid equation can be realized only by using deterministic DOF without solving the grid DOF of the reference region. The direct field dynamic stiffness matrix associated with subsystem j is determined by Eq. (42). This method can greatly reduce the scale of the hybrid surface connection matrix.

## Modification method of hybrid FE-SEA model

### The existing hybrid model connection correction method

**Figure 8:**
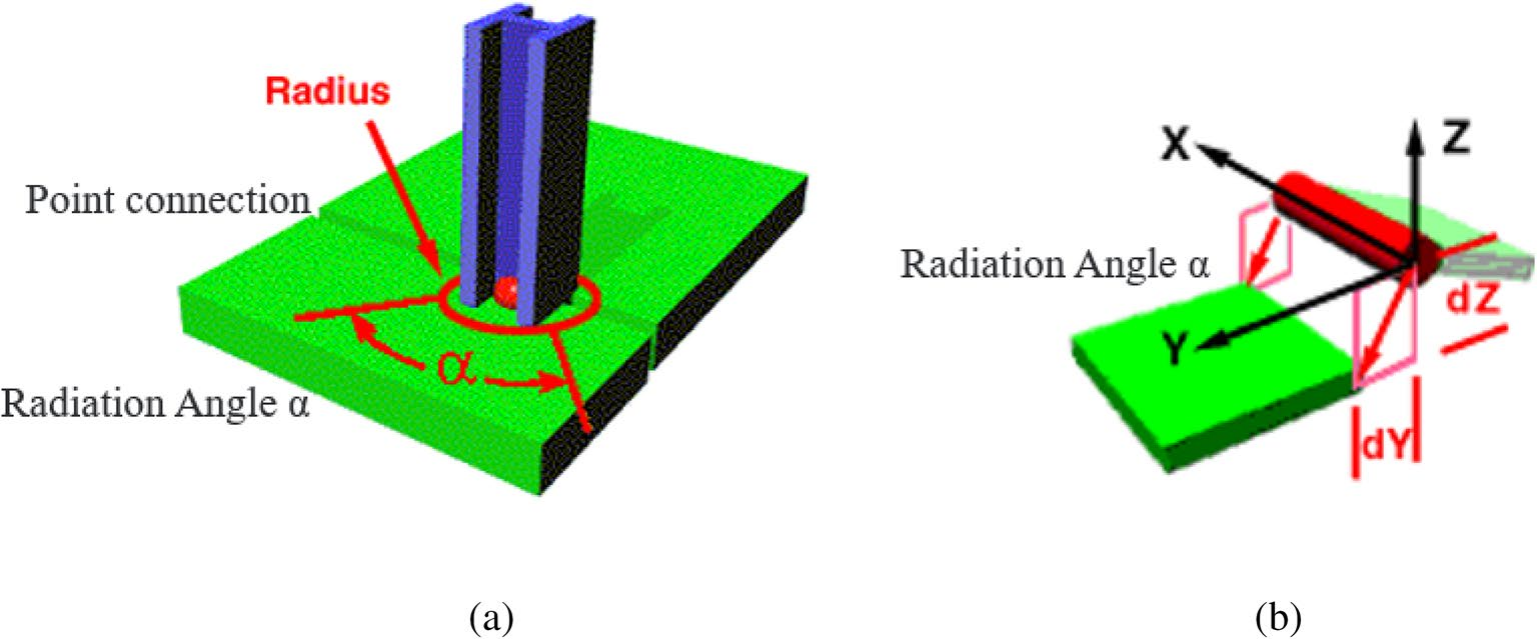
Schematic diagram of radiation Angle correction.

Based on the wave theory and the assumption of infinite thin plate structure, the boundary of the direct field dynamic stiffness matrix of point-line connection in the hybrid model needs to be evaluated by parameter correction. Generally, the modified radiation Angle α is used to describe the influence of point-line connection on boundary to boundary^6^, and the radiation Angle correction diagram is shown in Fig. 8. Figure 8a shows the radiation Angle diagram of the beam structure and the thin plate structure. The parameter α represents the radiation Angle at the point junction, and the connection relation attributes can be corrected by modifying the radiation Angle at the point junction. Figure 8b shows the schematic diagram of radiation Angle of line connection between thin plates. The radiation Angle between the two plates is α. X, Y and Z respectively represent the connection coordinate system at the line connection, and dz and dy represent the offset distance between the two thin plate structures and the line connection. The radiative impedance matrix of point-line connection in the hybrid model after parameter modification can be expressed as^20^:

54 $$D_{dir,\beta } = \beta_{\alpha } D_{dir,\infty } = (\alpha /2\pi )D_{dir,\infty }$$

where $\beta_{\alpha }$ is the correction factor of the point-line connection mode of the hybrid model. The subscript $\alpha$ represents the correction factor defined by the radiation Angle. Modifying the radiation Angle can modify the radiation impedance matrix of the point-line connection, so as to modify the precision of the point-line connection model. However, the radiation Angle correction method also has some limitations:

1)The radiation Angle parameter is defined as a constant term over the entire frequency range. The correction Angle with respect to frequency cannot be defined. It has certain limitations.

2)It is a difficult problem to determine the parameter range of radiation Angle when modeling the hybrid model with connection forms such as point and line. In the existing literature, the parameter selection range of radiation Angle has not been elaborated in detail.

### Modification method of hybrid point connection

Considering the displacement of the circular surface connected by points at ${\text{r}} = {\text{r}}_{{0}}$, the relation of displacement outside the plane in the polar coordinate system and the Cartesian coordinate system is expressed as follows:

55 $$u(r_{0} ,\theta ) = \omega + a\theta_{x} \sin \theta - a\theta_{y} \cos \theta$$

When ${\text{r}} = {\text{r}}_{{0}}$ it is assumed that the coefficients of related terms of $\theta$ are equal, and the following can be obtained^20,21^:

56 $$\omega = a_{{_{0} }}^{(b)} H_{0}^{(2)} (k_{b} r_{0} ) + a_{0}^{(bn)} H_{0}^{(bn)} ( - ik_{b} r_{0} )$$

57 $$\overline{\theta }_{x} = \frac{1}{{r_{0} }}[a_{{_{1s} }}^{(b)} H_{1}^{(2)} (k_{b} r_{0} ) + a_{1s}^{(bn)} H_{1}^{(2)} ( - ik_{b} r_{0} )$$

58 $$\overline{\theta }_{y} = \frac{1}{{r_{0} }}[a_{{_{1c} }}^{(b)} H_{1}^{(2)} (k_{b} r_{0} ) + a_{1c}^{(bn)} H_{1}^{(2)} ( - ik_{b} r_{0} )$$

Assuming that there is no relative sliding between the thin plate and the connecting circular surface, and assuming that the correlation coefficients of $\theta$ are equal, three displacement coordination conditions expressed by plane wave coefficients are obtained^21^:

59 $$a_{1c}^{(b)} [k_{b} H_{1}^{(2^{\prime})} (k_{b} r_{0} ) - (1/r_{0} ) - H_{1}^{(2)} (k_{b} r_{0} )] - a_{1c}^{(bn)} [ik_{b} H_{1}^{(2^{\prime})} (k_{b} r_{0} ) + (1/r_{0} )H_{1}^{(2)} ( - ik_{b} r_{0} )] = 0$$

60 $$a_{1s}^{(b)} [k_{b} H_{1}^{(2^{\prime})} (k_{b} r_{0} ) - (1/r_{0} ) - H_{1}^{(2)} (k_{b} r_{0} )] - a_{1s}^{(bn)} [ik_{b} H_{1}^{(2^{\prime})} (k_{b} r_{0} ) + (1/r_{0} )H_{1}^{(2)} ( - ik_{b} r_{0} )] = 0$$

61 $$k_{b} a_{0}^{(b)} H_{0}^{(2^{\prime})} (k_{b} r_{0} ) - ik_{b} a_{0}^{(bn)} H_{0}^{(2^{\prime})} ( - ik_{b} r_{0} ) = 0$$

Projecting the in-plane displacement in polar coordinates to the Cartesian coordinate system, the following relation can be obtained:

62 $$u = a_{1c}^{(e)} H_{1}^{(2^{\prime})} (k_{e} r_{0} ) + (1/k_{s} r_{o} )a_{1s}^{(s)} H_{1}^{(2)} (k_{s} r_{0} )$$

63 $$u = a_{1s}^{(e)} H_{1}^{(2^{\prime})} (k_{e} r_{0} ) + (1/k_{s} r_{o} )a_{1c}^{(s)} H_{1}^{(2)} (k_{s} r_{0} )$$

64 $$\theta_{z} = - (1/r_{0} )a_{0}^{(s)} H_{0}^{(2^{\prime})} (k_{s} r_{0} )$$

65 $$- a_{1s}^{(e)} [H_{1}^{(2^{\prime})} (k_{e} r_{0} ) - (1/k_{e} r_{0} )H_{1}^{(2)} (k_{e} r_{0} )] + a_{1c}^{(s)} [(1/k_{s} r_{0} )H_{1}^{(2)} \left( {k_{s} r_{0} } \right) - H_{1}^{(2^{\prime})} (k_{s} r_{0} )] = 0$$

66 $$- a_{1c}^{(e)} [H_{1}^{(2^{\prime})} (k_{e} r_{0} ) - (1/k_{e} r_{0} )H_{1}^{(2)} (k_{e} r_{0} )] + a_{1s}^{(s)} [(1/k_{s} r_{0} )H_{1}^{(2)} \left( {k_{s} r_{0} } \right) - H_{1}^{(2^{\prime})} (k_{s} r_{0} )] = 0$$

**Figure 9:**
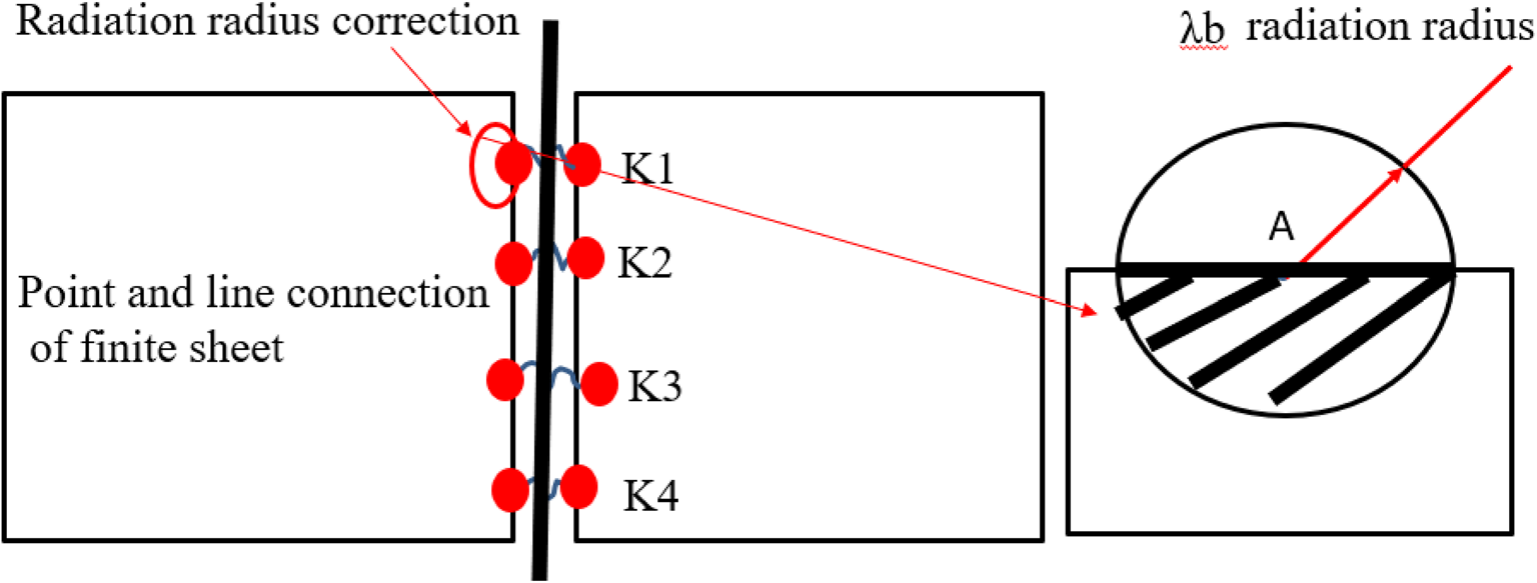
Radiation radius correction diagram.

Based on the propagation characteristics of structural waves in thin plates, a correction factor for the radiation radius of point connection is constructed. The hybrid model of point connection is modified by the correction factor of radiation radius. This method can effectively model and analyze the influence of connection boundary, so as to improve the analysis accuracy. As shown in Fig. 9, the bounded thin plate structure radiates energy to the thin plate structure through the wave number $k_{b}$ of the point connection. According to the thin plate theory, the structure's bending wave number $k_{b}$ is expressed as:

67 $$k_{b} = (ph/k_{w} )^{1/4} (2\pi f)^{1/2}$$

where $f$ is the frequency of the system, $p$ is the density of the thin plate, $h$ is the thickness of the thin plate, and $k_{w}$ is the bending stiffness. Further, the wavelength $\lambda_{b}$ in the thin plate structure system can be deduced, which can be expressed as:

68 $$\lambda_{b} = 2\pi /k_{b}$$

The point connection position is taken as the center of the circle, and the wavelength $\lambda_{b}$ of the thin plate structure is taken as the radiation correction radius to establish a circular region. The area where the thin plate structure region overlaps with the circular region is defined as the correction region of the point connection under the condition of frequency f. The correction factor of the radiation radius can be expressed as:

69 $$\beta_{i} = \frac{S}{{\pi \lambda_{b}^{2} }} = S\frac{{f_{i} }}{{2\pi^{2} }}\sqrt {\frac{ph}{{k_{w} }}} { + }\varepsilon_{i}$$

70 $$\varepsilon_{i} { = }\sum\limits_{k = 1}^{N} {\left| {\overset{\lower0.5em\hbox{$\smash{\scriptscriptstyle\frown}$}}{\beta }_{i} - \beta_{i} } \right|}$$

71 $$\overset{\lower0.5em\hbox{$\smash{\scriptscriptstyle\frown}$}}{\beta } = \frac{{\overset{\lower0.5em\hbox{$\smash{\scriptscriptstyle\frown}$}}{D}_{dir,\lambda } }}{{\overset{\lower0.5em\hbox{$\smash{\scriptscriptstyle\frown}$}}{D}_{dir,\infty } }}$$

where $$\overset{\lower0.5em\hbox{$\smash{\scriptscriptstyle\frown}$}}{\beta }$$ is the ratio of the dynamic stiffness matrix solved by the finite element method, which is used to correct the error term. Therefore, the direct field dynamic stiffness matrix of the modified hybrid connection based on the radiation radius is expressed as:

72 $$D_{dir,\lambda } = \beta_{i} D_{dir,\infty }$$

The correction factor of the radiation radius of the hybrid connection is related to the location of the hybrid connection, the analysis frequency and the inherent physical properties of the thin plate structure.

### Modification method of hybrid line connection

The dynamic equation was established based on the wavenumber space coordinate system. The direct field dynamic stiffness matrix of the coordinate system of nodes is obtained by Fourier transform. Let the displacement $\omega (x) = \sum\limits_{n} {a_{n} } u_{n} (x)$, where n = 1,2… N, $u_{n} (x)$ is a set of shape function values. Then the force generated by the node motion $\omega$ at the connection position x can be expressed in the convolution form^21,22^:

73 $${\text{f(}}x{) = }\int\limits_{L} {d(x - x^{\prime } )} \omega (x^{\prime } )dx$$

where $d(x - x^{\prime } )$ represents Green's function of the line connection model. Its physical meaning is the force generated at x by applying a unit pulse displacement at position $x^{\prime }$. Substitute the shape function $\omega (x) = \sum\limits_{n} {a_{n} } u_{n} (x)$ into Eq. (73), and the following relation can be obtained:

74 $${\text{f(}}x{) = }\sum\limits_{n} {a_{n} } \int\limits_{L} {d(x - x^{\prime } )} u_{n} (x^{\prime } )dx$$

The load obtained by Eq. (74) is projected onto the generalized coordinate $a_{m}$. Then the generalized force under the projection can be expressed as:

75 $$f_{m} = \int\limits_{L} {f(x)} u_{m} (x)dx$$

Substituting Eq. (74) into Eq. (73), the following relation can be obtained:

76 $$f_{m} = \sum\limits_{n} {a_{n} } \iint\limits_{L} {d(x - x^{\prime } )}u_{n} (x^{\prime } )u_{m} (x)dxdx^{\prime }$$

Therefore, the dynamic stiffness at the line connection is defined as:

77 $$D_{mn} = \iint\limits_{L} {d(x - x^{\prime } )}u_{m} (x)u_{n} (x^{\prime } )dxdx^{\prime }$$

According to the above equation, it can be deduced:

78 $$f_{m} = \sum\limits_{n} {D_{mn} a_{n} }$$

$u_{n} (x{\prime} )$ and $d(x)$ are expressed in inverse Fourier transform form:

79 $$u_{n} (x) = \frac{1}{2\pi }\int\limits_{R} {U_{n} } (k)e^{{_{{^{ikx} }} }} dk$$

80 $$d(x) = \frac{1}{2\pi }\int\limits_{R} D (k)e^{{_{{^{ikx} }} }} dk$$

where the value range of R is $\left\{ { - \infty \le K \le + - \infty } \right\}$. Substitute Eqs. (79) and (80) into the expression of $D_{mn}$ and the following relationship can be obtained^22^:

81 $$D_{mn} = \frac{1}{2\pi }\int\limits_{R} D (k)U_{m}^{*} (k)U_{n} (k)dk$$

**Figure 10:**
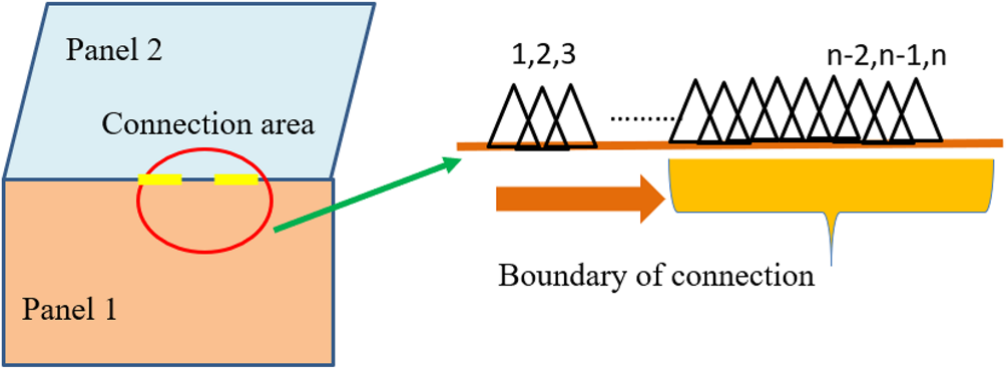
Graph of displacement function of line connection.

**Figure 11:**
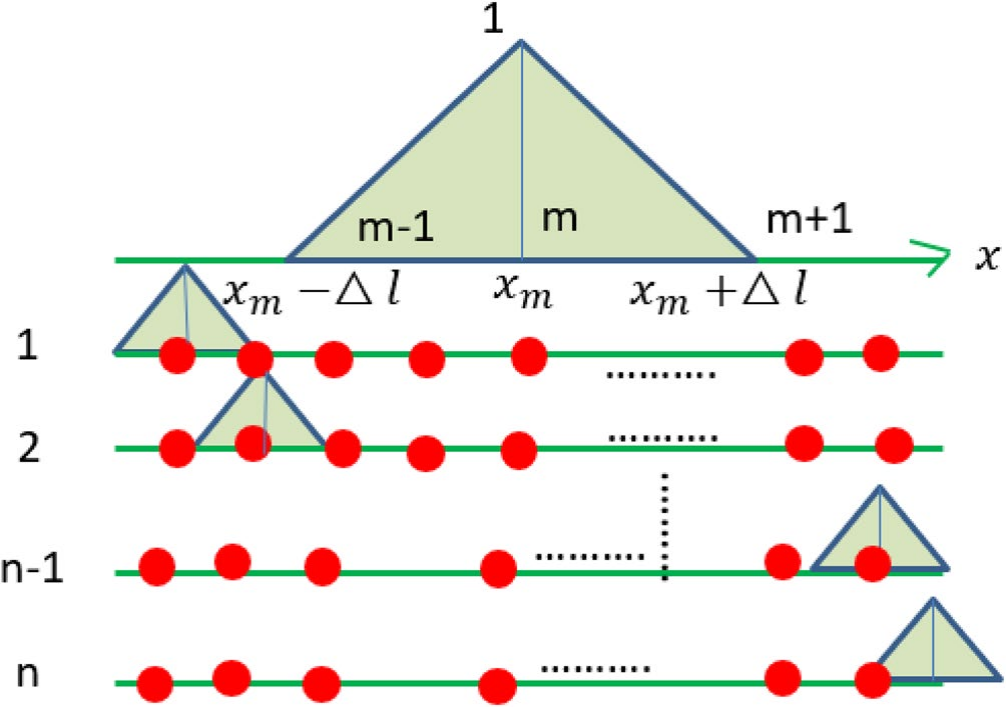
Shape function of the line connection.

The shape function of the linearly connected triangular wave is further determined. The triangular waveform function obtained by linear interpolation is shown in Fig. 10. The lateral displacement at the line connection can be expressed as:

82 $$\varpi (x) = \sum\limits_{n} {a_{n} u_{n} } (x)$$

where $u_{n} (x)$ is the nth shape function, and $a_{n}$ is the participating factor of the shape function. The node displacement can be solved by linear interpolation. The mth shape function is defined as follows: the displacement of the (m-1)th node and the (m + 1)th node is zero, and the intermediate displacement is calculated by linear interpolation. Then, the triangular waveform function as shown in Fig. 11 is constructed, and its function expression is as follows:

83 $$\phi_{m} (x) = \left\{ {\begin{array}{*{20}c} {1 - \frac{{\left| {x - x_{m} } \right|}}{\Delta l},} & {\left| {x - x_{m} } \right| \le \Delta l} \\ 0 & {\left| {x - x_{m} } \right| \succ \Delta l} \\ \end{array} } \right\}$$

where $\Delta l$ is the distance between nodes of the line connection model. The hybrid line connection model is modified by modifying the triangle wave function of the line connection in the hybrid model. Line connection correction of the hybrid model was carried out according to the empirical parameters of line connection of the shape function in Eq. (83)^20^. The direct field dynamic stiffness matrix of the model is modified by modifying the shape function of the hybrid line connection. The Fourier transform of the shape function of the node at x = 0 in wavenumber space is expressed as:

84 $$U_{(k)} = \int\limits_{ - \infty }^{ + \infty } {u(x)} e^{ - ikx} dx = \Delta l*{\text{Sin}} c^{2} \left( {\frac{k*\Delta l}{2}} \right)$$

where ${\text{Sin}} c(x) = \sin (x)/x$, for the shape function of any node, the expression of wavenumber space can be obtained by Fourier transform:

85 $$u_{n} (x) = u(x - x_{n} ) \to U_{n} (k) = \Delta l*{\text{Sin}} c^{2} \left( {\frac{k\Delta l}{2}} \right)e^{ - ikxn}$$

The shape function above is used to modify the parameters to establish the motion equation of the thin plate, and the displacement can be expressed as:

86 $$w = \left[ {\begin{array}{*{20}c} \omega \\ \theta \\ \end{array} } \right] = \sum\limits_{n} {a_{n} } \phi_{n} = \sum\limits_{n} {\left[ {\begin{array}{*{20}c} {a_{n} } & 0 \\ 0 & {b_{n} } \\ \end{array} } \right]} \left[ {\begin{array}{*{20}c} {u_{n} (x)} & 0 \\ 0 & {u_{n} (x)} \\ \end{array} } \right]$$

According to the above shape function, the dynamic stiffness matrix of the direct field connected by the line in the node coordinate system can be obtained:

87 $$D_{dir,mn} = \frac{1}{2\pi }\int\limits_{L} {\left[ {\begin{array}{*{20}c} {D_{11} (k)} & {D_{12} (k)} \\ {D_{21} (k)} & {D_{22} (k)} \\ \end{array} } \right]} *\left[ {\begin{array}{*{20}c} {U(k)^{2} } & 0 \\ 0 & {U(k)^{2} } \\ \end{array} } \right]e^{ - ik} (x_{n} - x_{m} )dk$$

## Algorithm verification analysis

### Validation analysis of hybrid point connection correction method

**Figure 12:**
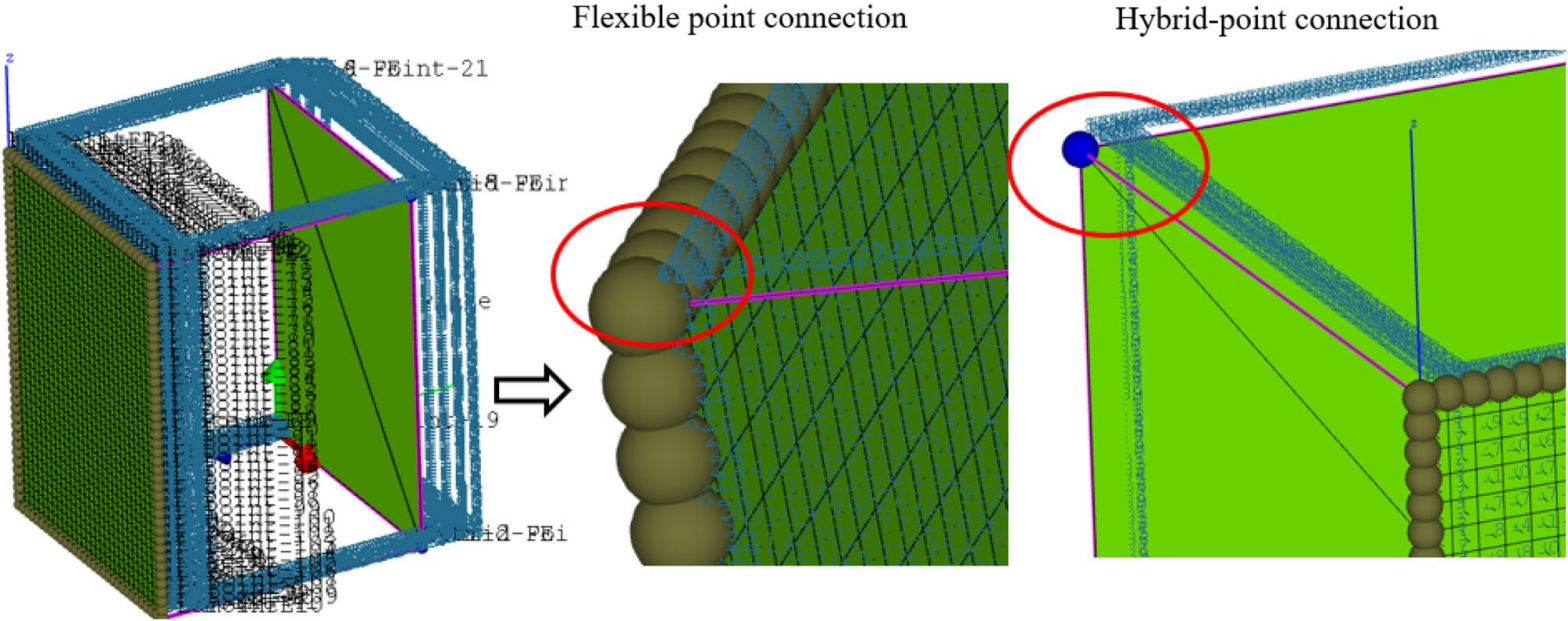
Hybrid FE-SEA model.

**Figure 13:**
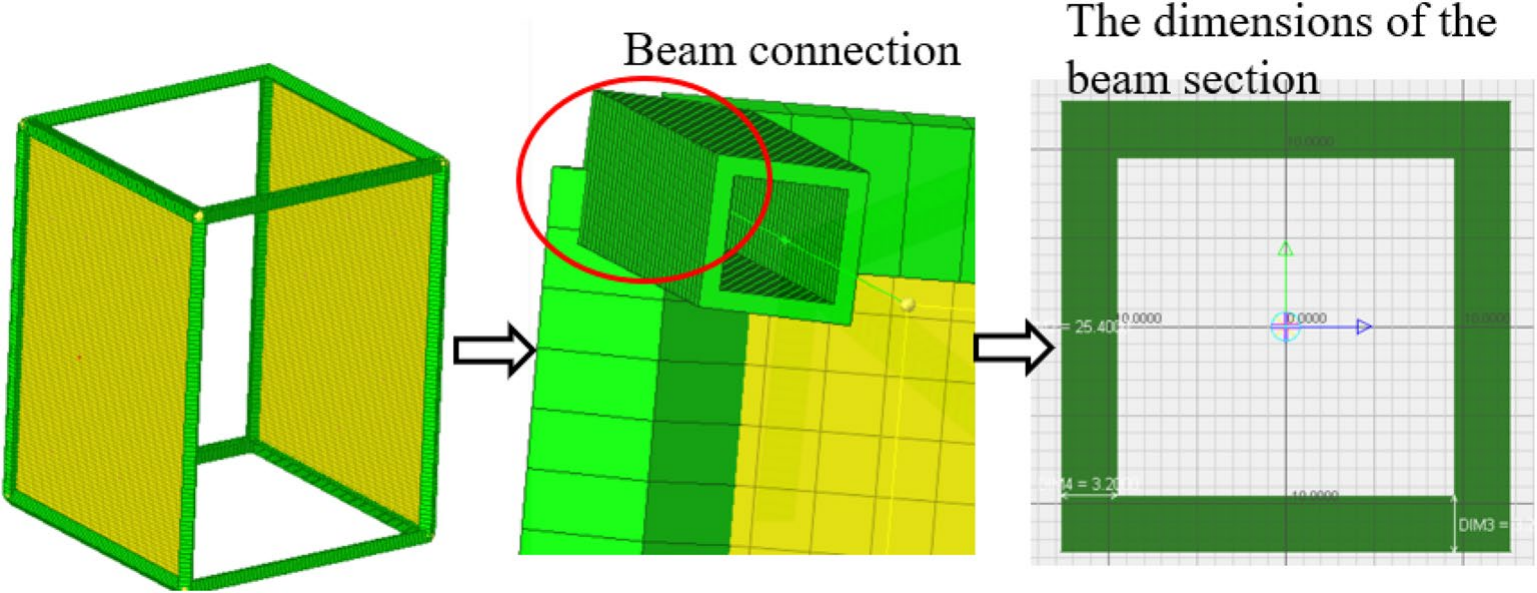
Finite element model.

In order to verify the validity of the correction factor model of point connection, the structure shown in Fig. 12 was analyzed. The model is composed of a beam structure and two thin plate structures. The material properties of the hybrid model are shown in Table 1. The thin plates are connected to the beam structure through joints. The material of the beam structure is steel, the cross section is hollow square, the height of the beam is 25.4 mm, the thickness of the beam is 3.2 mm, and the combined structure is composed of 12 beams. The structure size of the thin plate is 600 mm × 800 mm, the thickness of the thin plate is 1 mm, the material is aluminum, and the analysis frequency is 1–1300 Hz. Loads are applied laterally to one of the plate structures. Since the point connection is located inside the thin plate structure, the radiation Angle (360°) of the point connection under ideal conditions is converted to a correction factor of 1. The connection method of the model is modeled by the hybrid point connection model. Then the radiation radius correction factor is used to correct it. In the process of hybrid model modeling, the in-plane motion of the thin plate structure and the beam structure are used to (FEA) determine the structural modeling. The out- plane motion of the thin plate is modeled by the statistical energy (SEA) due to the relatively small wavelength. Figure 13 shows the finite element model of the structure. Considering the uncertainty of the medium frequency response of the hybrid model, the finite element energy flow method and Monte Carlo test method were used as the reference solution of the numerical simulation. The method is used to simulate the perturbation of structural parameters by applying 20 random distributed masses on each thin plate with a total mass of 15%. The radiation radius method proposed in this paper is compared with the radiation Angle method of the existing technology method. The radiation Angle is selected by the setting method in Literature^22^. The radiation angles were 360°, 180° and 90°, respectively. According to the reference^20^ and Eq. (69), the equivalent correction factors were β = 1, β = 0.5 and β = 0.25, respectively.

**Table 1:** Hybrid model material parameters.

Name	Density (kg/m^3^)	Poisson’s ratio	Elastic model (GPa)	Damping loss factor	Structure size (mm)	Structural thickness (mm)
Beam	7800	0.30	200	0.01	25.4	3.2
Aluminium plate	2700	0.33	70	0.01	600 × 800	1

**Figure 14:**
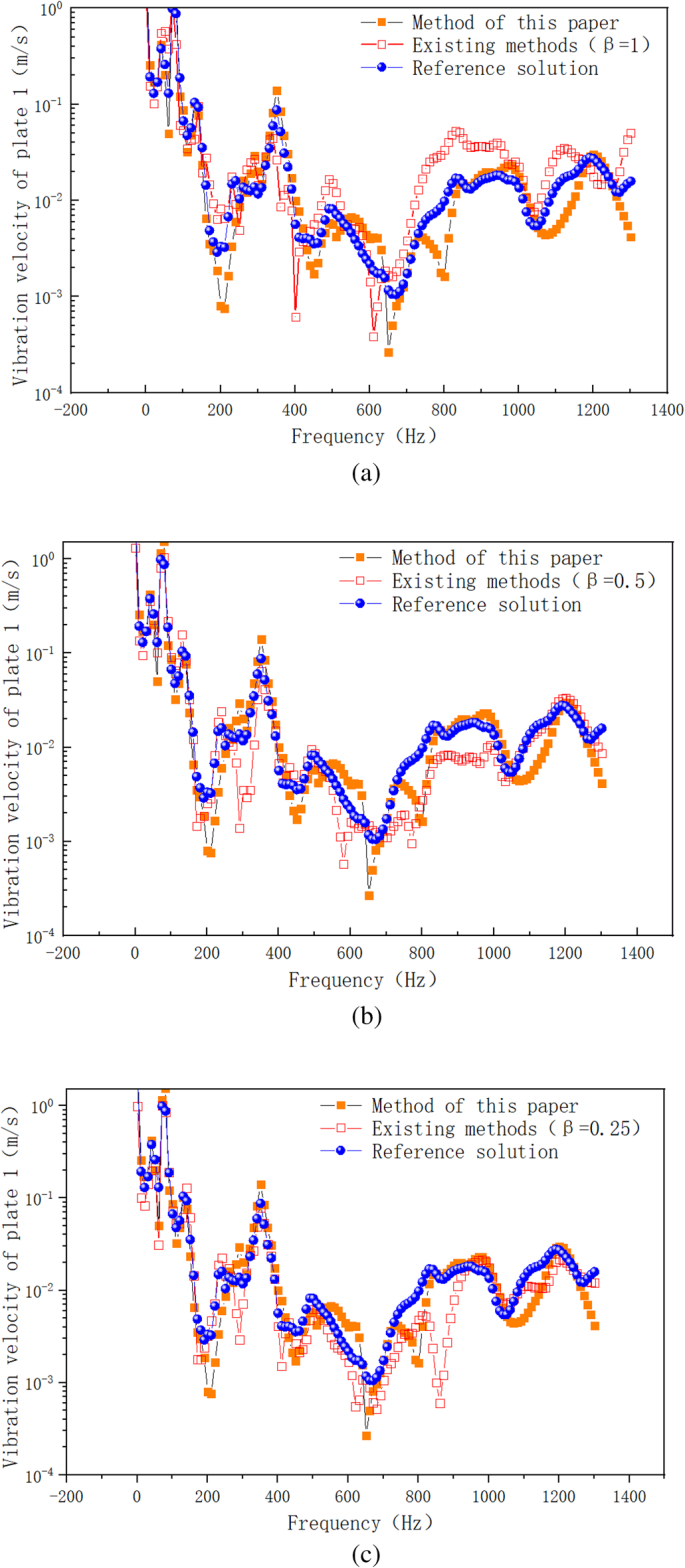
Vibration velocity of plate 1. (**a**) Comparison of the improved method with the existing method (β = 1). (**b**) Comparison of the improved method with the existing method (β = 0.5). (**c**) Comparison of the improved method with the existing method (β = 0.25).

**Figure 15:**
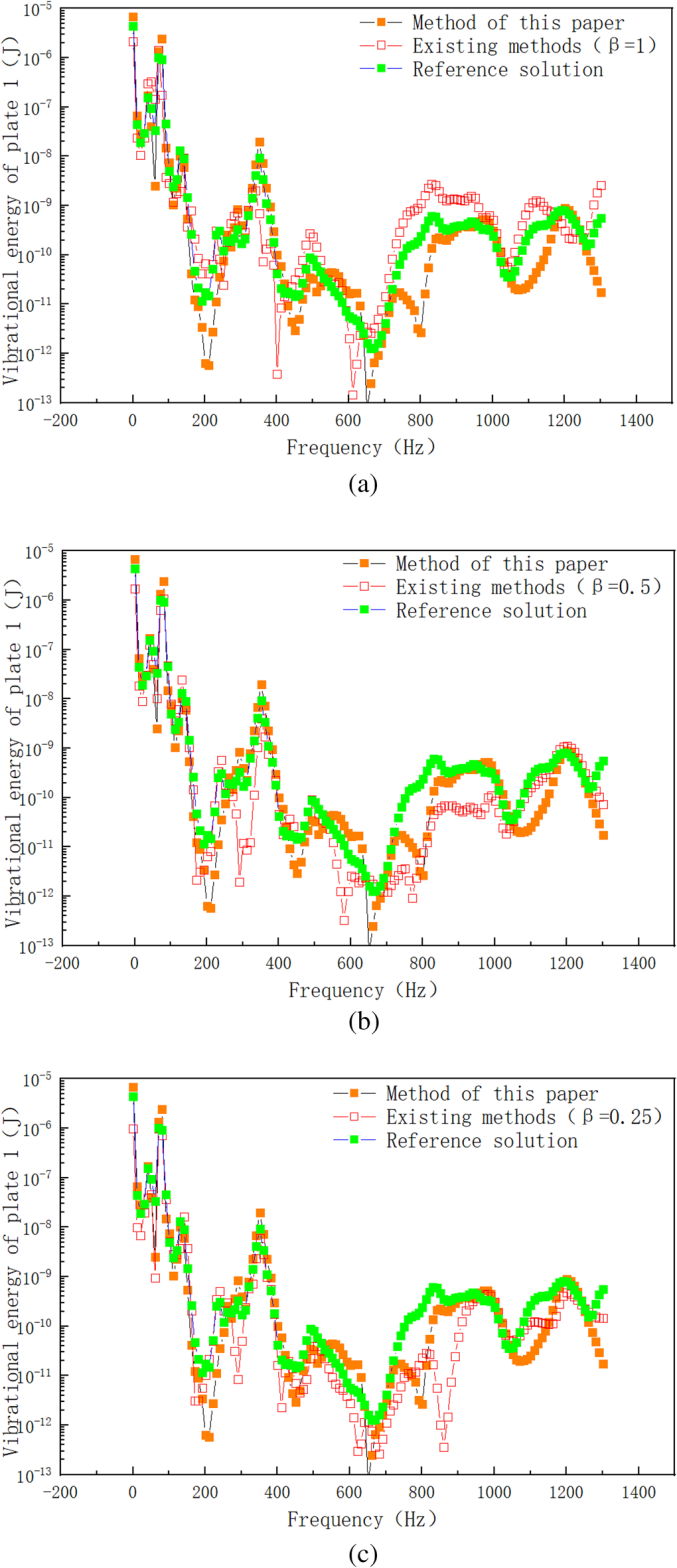
Vibration energy of plate 1. (**a**) Comparison of the improved method with the existing method (β = 1). (**b**) Comparison of the improved method with the existing method (β = 0.5). (**c**) Comparison of the improved method with the existing method (β = 0.25).

Figure 14 shows the numerical simulation results of vibration velocity of plate 1 by different calculation methods. As can be seen from Fig. 14a, the error between the existing method (β = 1) and the reference solution is large in the frequency range of 800–1200 Hz, and the maximum error occurs in the frequency range of 800–1000 Hz. The proposed method is used to modify the point connection modeling, and the fitting degree between the modified curve and the reference solution is good in the whole frequency band. Within the range of 0–600 Hz, the method in this paper is basically consistent with the curve of reference solution. Within the frequency range of 800–1200 Hz, the error range between the proposed method and the reference solution is also within the reasonable engineering error range. The peak correspondence of the curve is good in the whole frequency range, which indicates that the correction factor method based on the radiation radius is more suitable for the correction of the intermediate frequency response of the hybrid model than the radiation Angle method of the prior art. Compared with the existing literature^20^, the correction accuracy of the proposed method for the whole frequency band is higher, and it is suitable for the point connection model correction of hybrid FE-SEA model. Compared with the reference^20^, the hybrid model point connection correction method proposed in this paper is based on the existing theory. Wavelength based error correction factor is introduced into the point correction factor, and the radiation radius error is corrected through wavelength error correction, so as to improve the calculation accuracy. Based on the reference^20^, the method of this paper fully considers the error amount of the correction factor, and makes a mathematical correction to the error amount of the correction factor. Figure 14b,c show the comparison between the existing method (β = 0.5, β = 0.25) and the proposed method. It can be seen from the correction results that although the radiation Angle correction by the existing method can reduce the response error of the whole system, the correction accuracy is still far from the method presented in this paper. In order to further verify the differences between the proposed method and the existing methods, Fig. 15 shows the numerical calculation results of vibration energy of plate 1 by different calculation methods. From Fig. 15a–c, it can be seen that the error between the existing method (β = 1, β = 0.5, β = 0.25) and the reference solution is large in the intermediate frequency range. The modified method of radiation radius in this paper is in good agreement with the Monte Carlo reference solution. In the low frequency range of 0–600 Hz, the proposed method has a high degree of curve fit with the reference solution. The peak-peak correspondence of the curve is good in the whole low frequency range. In the intermediate frequency range of 600–1200 Hz, the method presented in this paper is consistent with the peak trend of the reference solution curve. The peak-peak value is within a reasonable error range, and the simulation accuracy is higher than that of the existing methods. In this paper, the radius-based mixing point connection model modification method can be used as a mixing model modification method, which provides a solution for accurate mixing model modeling and modification.

### Validation analysis of hybrid line connection correction method

**Figure 16:**
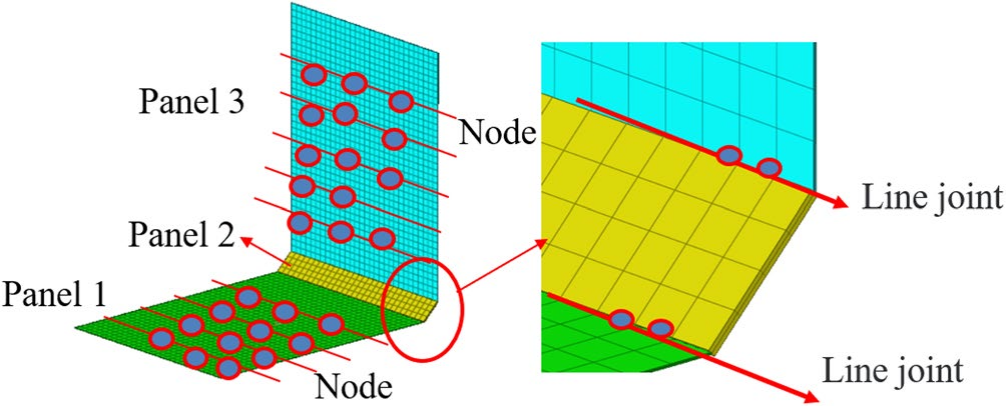
Line connection finite element model.

**Figure 17:**
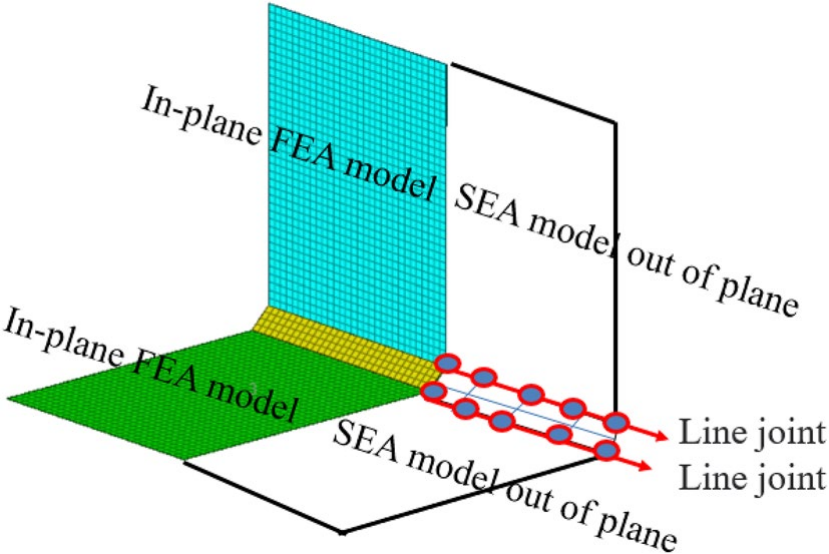
Hybrid line connection model.

The line connection of the hybrid model can be modified by modifying the shape function of the line connection. In order to verify the effectiveness of the modified hybrid line connection model, the structure shown in Fig. 16 was verified. The thin plate structure of the hybrid line connection model is composed of two plate elements of the same size and connecting plate. Its material parameters are shown in Table 2. The sheet material is aluminum sheet, with a length of 600 mm, a width of 700 mm and a thickness of 1 mm. The connecting plate is made of steel structure, with the length of 600 mm, width of 70 mm and thickness of 4 mm. The analysis frequency range is 0–1000 Hz. Apply unit sweep excitation on plate 1. 20 nodes were randomly selected from each thin plate as Montecarlo response reference points^20^ to solve the vibration velocity and coupling loss factor of the system. Considering the dynamic characteristics of the whole system, the displacement out-plane with high modal density is modeled by the statistical energy method. The finite element model is used for the joint plates with small modal density and large in-plane displacement and stiffness. The two hybrid models are coupled through two hybrid lines, and the hybrid model is shown in Fig. 17.

**Table 2:** Hybrid model material parameters.

Name	Density (kg/m^3^)	Poisson’s ratio	Elastic model (GPa)	Damping loss factor	Structure size (mm)	Structural thickness (mm)
Steel plate	7800	0.30	200	0.01	600 × 70	4
Aluminium plate	2700	0.33	70	0.01	600 × 700	1

**Figure 18:**
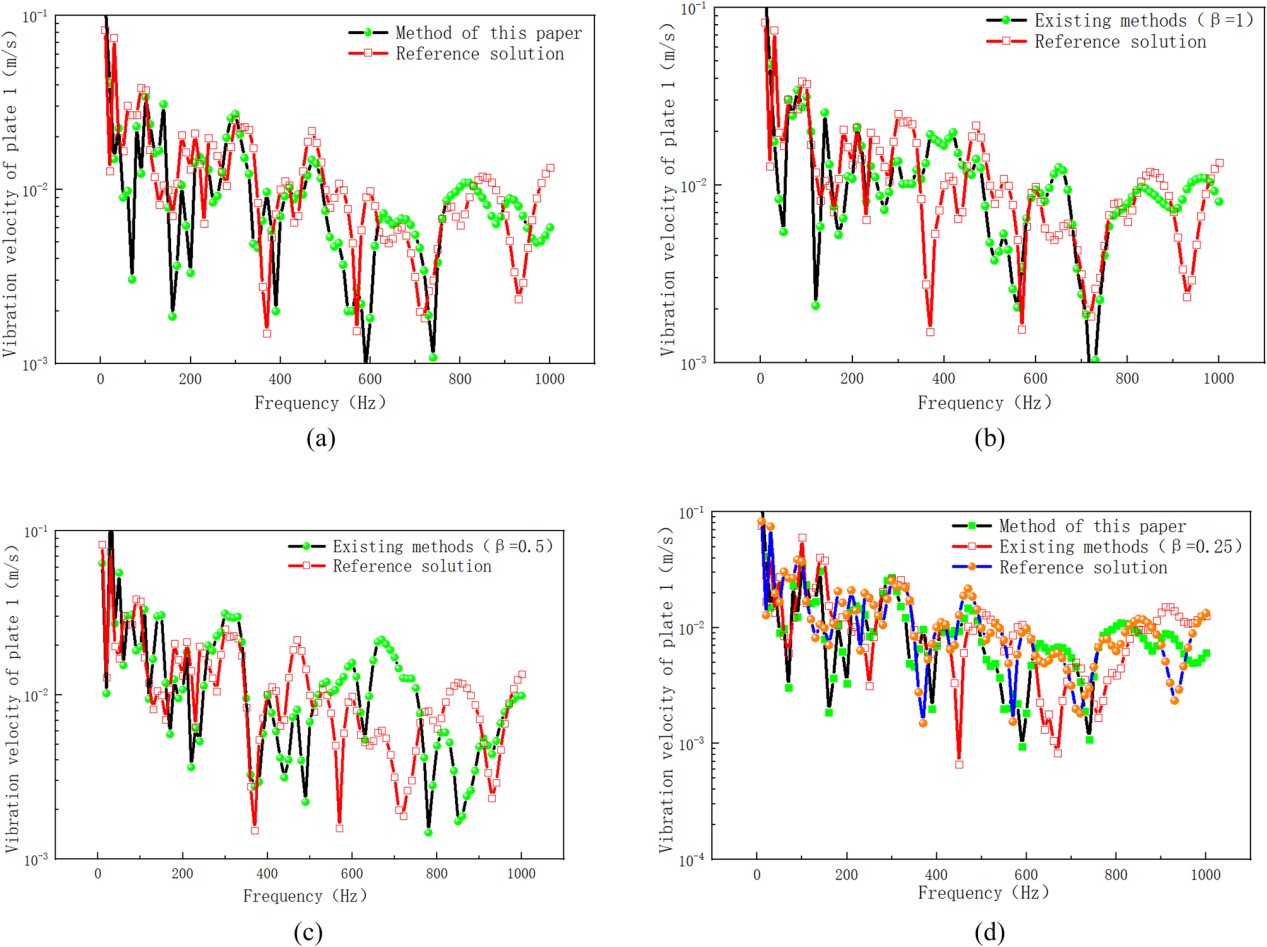
The vibration velocity calculated by different methods.

The reference solution is a hybrid finite element energy flow method combined with Monte Carlo test. The finite element method is a widely used dynamic analysis method at present. At the same time, Monte Carlo simulation is used to introduce parameter perturbation to describe the influence of uncertainty on intermediate frequency response. Since the finite element method does not use a lot of assumptions, the results of the energy flow analysis-Monte Carlo simulation method can be used as the basis for improving the statistical energy method or the prediction method of the medium frequency mechanical environment. In the existing methods, the setting method in the literature^22^ is used to correct the radiation Angle, and the radiation Angle is 360°, 180° and 90° respectively. According to the literature^20^ and Eq. (69), the equivalent correction factors are β = 1, β = 0.5 and β = 0.25, respectively. Figure 18 shows the vibration velocity curves of plate 1 calculated by different methods. As can be seen from Fig. 18a, the curve fitting degree between the proposed method and the Montecarlo reference solution is relatively high in the frequency range of 0–1000 Hz. The peak-peak correspondence of the curve is good in the frequency range of 0–500 Hz, and the maximum error of the whole frequency band is less than 10^–1^. Compared with literature^20,21^, the method in this paper has a higher degree of curve fitting in the middle frequency band. Figure 18b shows the comparison between the existing method (β = 1) and the reference solution. It can be seen from the figure that the existing method (β = 1) has a large error with the reference solution in frequency bands of 100 Hz, 370 Hz, 700 Hz, etc., and the peak-peak correspondence of the curve is poor. Similarly, the existing methods (β = 0.5, β = 0.25) in Fig. 18c,d have large errors in multiple peak frequencies with the reference solution, and the curve fitting degree is not high.

**Figure 19:**
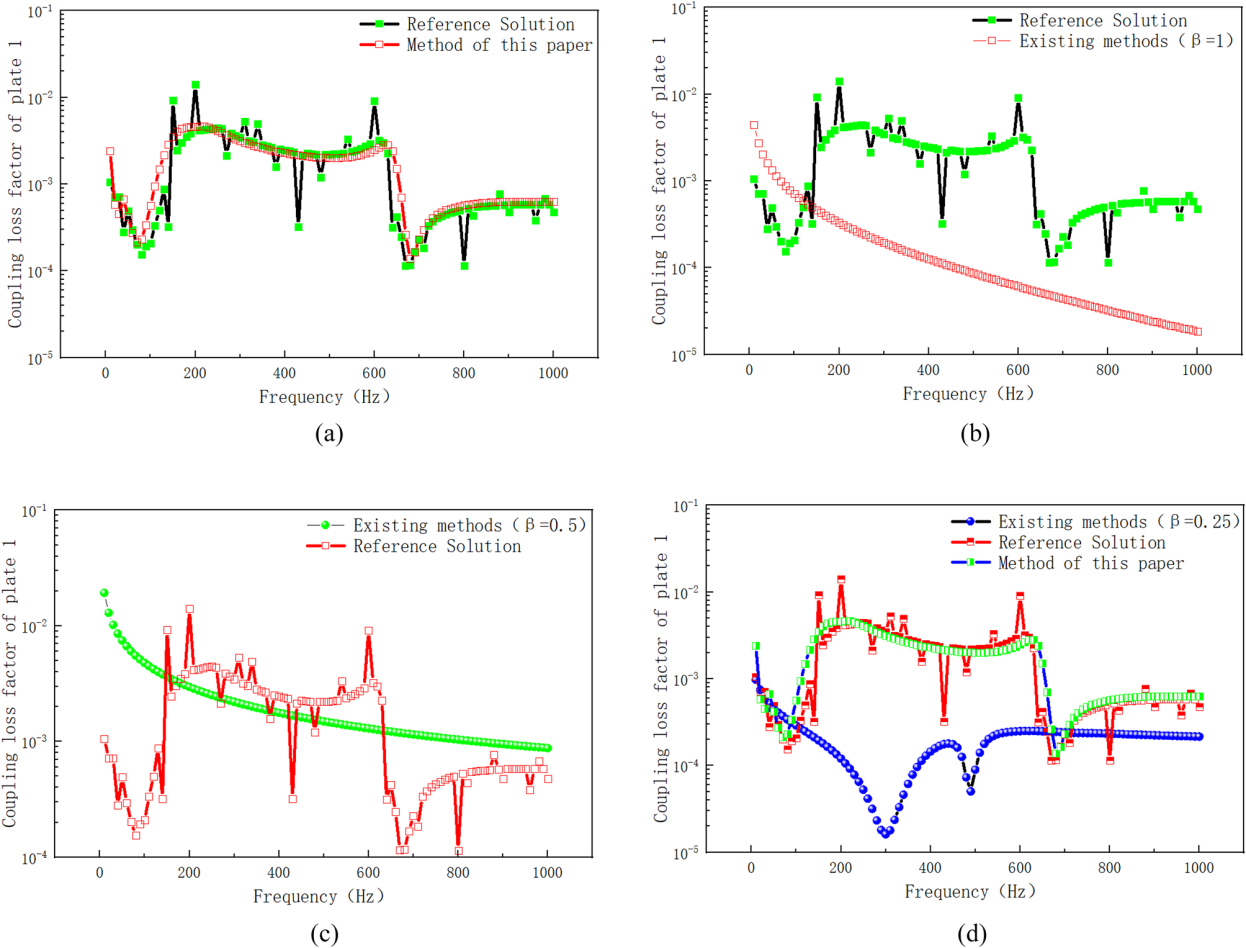
The coupling loss factor of different calculation methods.

In order to further verify the modified method of hybrid line connection, the coupling loss factor is verified for the hybrid model of line connection. Figure 19 shows the calculation results of coupling loss factors of plate 1 with different calculation methods. It can be seen from Fig. 19a that the proposed method has a high degree of fit with the Montecarlo reference solution. In the frequency range of 0–500 Hz, the peak value correspondence of the coupling loss factor curves obtained by the proposed method and the reference solution method is good, and the maximum error is less than 10^–1^, which shows a high precision of linear connection calculation. When the frequency range is 500–1000 Hz, the other frequencies have good calculation accuracy except for the weak fluctuation of individual peaks. Compared with reference^20,21^, the modeling and correction accuracy of hybrid line connection using the method in this paper can effectively improve the calculation accuracy of Intermediate Frequency and the corresponding frequency of peak-peak value of curve. Based on reference^20,21^, this paper synthesizes the existing theoretical methods of radiation Angle correction and shape function correction. The conversion method of radiation Angle is optimized, the modified method of mixed line connection based on form function is optimized, and the node form function and the node dynamic matrix based on form function are optimized, which are further optimization of the existing theoretical methods. Figure 19b is a calculation comparison between the existing method (β = 1) and the reference solution. It can be seen from the figure that the existing method (β = 1) has poor correspondence with the trend of the reference solution curve, and the peak error in the whole calculated frequency band is large. Similarly, Fig. 19c,d show a comparison between the existing method (β = 0.5, β = 0.25) and the reference solution. It can be seen from the figure that the correction error of the line connection model of the radiation Angle in the existing method is large, and it is not consistent with the curve of the reference solution, and the peak-peak frequency does not have good correspondence in the whole calculated frequency band. The above numerical examples show that the existing methods have large errors in the correction of the hybrid line model, and the selection range of radiation Angle correction by the existing techniques has certain limitations. The method in this paper is modified based on the shape function of the hybrid line connection model, so as to modify the direct field dynamic stiffness matrix at the joint. Compared with the existing methods, it has a good practical value and can provide a solution for the modification of hybrid wire connection model.

## Conclusion

In order to solve the problem of the modeling method and correction of the "hybrid-point" connection in the hybrid FE-SEA model, the “out-plane” tension wave and shear wave motion equations of the “hybrid-point” connection are established by using the superposition principle of plane waves in polar coordinates. The relation between “hybrid-point” connection and wave number in Cartesian coordinate system is studied. According to the wave propagation characteristics of thin plate structures, a “hybrid-point” connection radiation radius correction method is proposed. Compared with the prior art, the modification of the hybrid FE-SEA model with the radiation radius correction method is in good agreement with the Monte Carlo reference solution results. An example study shows that the error between the existing method (β = 1) and the reference solution is large in the frequency range of 800 Hz to 1200 Hz. The proposed method is used to modify the “hybrid-point” connection modeling, and the fitting degree between the modified curve and the reference solution is good in the whole frequency band. In the range of 0–600 Hz, the method in this paper is basically consistent with the curve of reference solution. In the frequency range of 800–1200 Hz, the error between the proposed method and the reference solution is within a reasonable range. Compared with reference^20,21^, the correction accuracy of the proposed method for the spectrum in the whole frequency band is higher.

In order to solve the modeling method and correction problems of the “hybrid-line” connection in the hybrid FE-SEA model, the triangular waveform function model of the “hybrid-line” connection was established by using the linear difference method. According to the “hybrid-line” connection shape function in wavenumeral space, a modification method of “hybrid-line” form function is proposed. The numerical example shows that the modified method based on the shape function has a good fit with the Monte Carlo reference solution. In the range of 0–500 Hz, the method presented in this paper has a good correspondence with the peak value of the coupling loss factor curve obtained from the reference solution, and the maximum errors are all less than 10^–1^. In the frequency range of 500–1000 Hz, except for the weak fluctuation of individual peaks, the other frequencies have good calculation accuracy. Compared with reference^20–22^, the accuracy of the “hybrid-line” connection modeling and correction is effectively improved.

## Data Availability

Te datasets used and/or analyzed during the current study are available from the corresponding author upon. reasonable request.
